# *Colla Corii Asini* regulate collagen regeneration in UV exposure-induced skin photoaging in mice

**DOI:** 10.1186/s13020-025-01175-1

**Published:** 2025-09-22

**Authors:** Yuman Ma, Jun Huang, Junxiao Gong, Lishuang Li, Yujia Zhao, Yucui Jin, Jianjun Gu, Haibin Liu, Yi Wang, Yanan Sun

**Affiliations:** 1 Experimental Research Center, China Academy of Chinese Medical Sciences, No.16, Nanxiao Street, Dongzhimen, Dongcheng District, Beijing, People’s Republic of China; 2 School of Traditional Chinese Materia Medica, Shenyang Pharmaceutical University, Shenyang, 110016 People’s Republic of China; 3 Dong’e Ejiao Co., Ltd., Liaocheng, 252200 China

**Keywords:** CCA, Peptides, Photoaging, Collagen synthesis, Collagen degradation

## Abstract

**Background:**

Ultraviolet (UV) radiation is the primary external factor driving skin aging, mainly due to imbalanced collagen synthesis and degradation. Identifying new drugs targeting collagen regeneration may effectively delay skin aging. *Colla Corii Asini* (CCA), derived from donkey skin, contains abundant proteins and peptides, suggesting potential benefits against photoaging through collagen homeostasis regulation. However, its mechanisms and active components remain unclear.

**Objective:**

This study aimed to evaluate whether CCA mitigates photoaging by regulating collagen synthesis and degradation, identifying its active components and molecular targets to support future pharmacological studies.

**Methods:**

A photoaging mouse model was established, and the therapeutic effects of CCA were assessed through in vivo optical imaging and ex vivo analyses. Human dermal fibroblasts were employed to explore mechanisms of collagen regulation by CCA. PPI analysis and AlphaFold3 were used to predict and validate key active peptides and molecular targets.

**Results:**

CCA significantly improved the structure and appearance of photoaged skin, reduced oxidative stress and inflammation, and enhanced collagen content and density. Mass spectrometry identified 98 peptides closely linked to protein synthesis. Mechanistically, CCA increased collagen synthesis and reduced aging markers by activating the TGFβ-SMAD signaling pathway, balancing MMPs and TIMPs. Importantly, this effect is primarily associated with the core CCA peptide YYTSASGDEMVSLK binding to and upregulating TGFBR1 and TGFBR2 expression, activating the TGFβ-SMAD pathway, and promoting collagen regeneration.

**Conclusion:**

CCA, particularly peptide YYTSASGDEMVSLK, has potential to slow skin photoaging, highlighting a promising therapeutic strategy for skin aging management.

**Supplementary Information:**

The online version contains supplementary material available at 10.1186/s13020-025-01175-1.

## Introduction

Counteracting photoaging has long been a significant challenge for the scientific community. Photoaging, a skin aging process caused by prolonged exposure to ultraviolet (UV) radiation, accounts for approximately 80% of skin aging [1–3]. Its main characteristics include skin thinning, wrinkles, irregular pigmentation, telangiectasia, and enlarged pores. These changes not only affect appearance but may also lead to severe conditions such as skin cancer, imposing a significant economic burden on patients and society [4]. UV radiation is categorized based on wavelength into UVA (320–400 nm), UVB (280–320 nm), and UVC (200–280 nm). Among them, UVA has strong penetration ability, reaching the dermis to directly damage elastic and collagen fibers, while also inducing the production of reactive oxygen species (ROS), exacerbating dermal fiber destruction. UVB has a shorter wavelength but higher energy, capable of causing DNA breaks in epidermal cells, triggering the release of inflammatory factors and oxidative stress molecules, ultimately leading to skin inflammation and tissue damage. UVC has minimal penetration ability and is almost entirely absorbed by the ozone layer, exerting negligible effects on human skin [5–8]. Therefore, photoaging is primarily the result of combined UVA and UVB exposure.

Currently, the main approaches to slow and prevent photoaging include physical protection, topical retinoic acid or antioxidant drugs, and medical cosmetic treatments [9, 10]. However, these methods still have certain limitations regarding stability and safety. In recent years, peptides extracted from various plant or animal proteins have demonstrated excellent bioactivity, high specificity, and low drug resistance, showing potential to repair or activate biological functions and improve photoaged skin [11–16]. Collagen is the primary component of the dermal extracellular matrix (ECM), accounting for 70% of the skin’s dry weight. It provides structural support, maintaining skin elasticity and firmness, and is mainly secreted by dermal fibroblasts [17]. During skin photoaging, UV radiation disrupts multiple molecular signaling pathways, disturbing the dynamic balance between the production and degradation of collagen fibers in the dermis. This reduction in collagen content leads to wrinkles, laxity, and roughness of the skin [18]. Therefore, considering the critical role of collagen in the initiation and progression of photoaging, exploring the potential of peptides to regulate imbalances in collagen regeneration is of significant research value.

CCA is an advanced traditional chinese medicine commonly used in China and widely applied in modern medicine for treating anemia, chronic fatigue syndrome, and immune-related diseases. In recent years, research on CCA has expanded to explore its potential roles in anti-aging and skincare. The primary components of CCA include proteins, peptides, and amino acids, with protein content ranging from 60 to 85%, primarily comprising type I and type III collagen [19, 20]. Oral administration of CCA delivers abundant collagen peptides and hydroxyproline, which can directly supplement collagen or stimulate fibroblast proliferation and collagen secretion to improve photoaged skin [21]. Studies have shown that enzymatically hydrolyzed CCA promotes the expression of type IV collagen and elastin in UV-induced human gingival fibroblasts and 3D skin models, reducing wrinkle severity in the 3D skin model [22]. Oxidative stress is one of the primary causes of skin photoaging. Studies have demonstrated that CCA enhances immune function and reduces oxidative stress by increasing antioxidant enzyme activity and scavenging free radicals [23]. However, despite its multi-target and multi-pathway pharmacological effects and significant potential in alleviating photoaging, specific mechanism and many peptides contained in CCA remain unexplored. Current studies on its key active components are largely limited to bioinformatics analyses, lacking experimental validation and detailed exploration of its mechanisms of action.

Therefore, this study aimed to investigate whether CCA could improve photoaging by establishing animal and cellular photoaging models. Through this study, we aim to elucidate the pharmacological effects and specific mechanisms of CCA in mitigating photoaging and further identify the bioactive components responsible for maintaining collagen fiber structure and linking them to efficacy. This consequently expands the therapeutic value and potential of CCA and provide scientific support for its further development in the field of photoaging prevention.

## Materials and methods

### Mass-spectrometric identification of CCA

CCA samples were analysed by zenoSWATH mass spectrometry on a SCIEX ZenoTOF 7600 instrument (507796, SCIEX, USA). After liquid-chromatographic separation, ions entered the mass spectrometer under positive-mode electrospray ionisation (ESI) at a spray voltage of 5500 V and a source temperature of 320 °C; nebuliser gas (GS1) and auxiliary gas (GS2) were set to 50 psi, and curtain gas (CUR) to 35 psi. Data were acquired in zenoSWATH mode with an MS survey range of m/z 400–1200 and an MS/MS range of m/z 100–1500. Q1 isolation windows were variable, and collision-induced dissociation (CID) energies were automatically optimised using the standard rolling energy equation. The Zeno trap was activated to enhance the capture efficiency of low-abundance ions. Peptides and proteins were searched against the Equus asinus UniProt database. Raw files were processed and quantified with DIA-NN, and peptide- and protein-level identifications were filtered to a 1% false-discovery rate (FDR).

### GO enrichment analysis, protein–protein interaction (PPI) and bioinformatics analysis

A total of 98 proteins, identified from 176 peptides by mass spectrometry, were mapped to mouse homologous genes using NCBI HomoloGene. GO enrichment analysis was performed using SRplot software. Proteins related to the TGFβ–SMAD pathway were retrieved from the KEGG database and mapped to mouse homologous genes via NCBI HomoloGene. The two sets of mouse homologous genes described above were uploaded to Metascape to construct a PPI network. The network was analyzed using default confidence scores from the STRING database, and modules were identified using the Molecular Complex Detection (MCODE) algorithm. The three-dimensional structure of the peptide YYTSASGDEMVSLK was predicted using the AlphaFold3 platform, and the local distance difference test (PLDDT) scores for its interaction with TGFBR1/TGFBR2 were evaluated.

### Animal modeling and drug administration

Forty 8-week-old male BALb/c mice (weight 18–20 g) (R510-22-16, RWD Life Science Co., Ltd., China) were housed in a controlled environment with a 12-h light/dark cycle, standard diet, and free access to water. The animal experiment was supervised and approved by the Ethics Review Committee of the Chinese Academy of Medical Sciences (ERCCACMS21-2412-03). The 40 mice were randomly divided into four groups, each containing 10 mice: control, model, CCA, and VC groups. After removing the hair from the dorsal region with depilatory cream, the dorsal skin was irradiated using a UV therapy device (SS-07, Shanghai SIGMA High-tech Co., Ltd, China) at a distance of 5 cm, five times per week for 9 weeks. The radiation doses were as follows: Week 1: UVA dose 0.6 J, UVB dose 60 mJ/cm^2^; Week 2: UVA dose 0.9 J, UVB dose 90 mJ/cm^2^; Week 3: UVA dose 1.2 J, UVB dose 120 mJ/cm^2^; From Week 4 onward: UVA dose 1.8 J, UVB dose 180 mJ/cm^2^, because individual photon energy differs between bands, UVA delivers lower energy and requires substantial cumulative exposure to elicit biological effects; experimental models therefore report UVA doses in J. Conversely, the higher energy UVB elicits pronounced responses at much lower exposures, so its doses are conventionally expressed in mJ/cm^2^ [24, 25]. Drug solutions were prepared using normal saline. CCA was dissolved by heating in a 60 °C water bath to a concentration of 150 mg/mL, while the VC solution was prepared at a concentration of 30 mg/mL. Mice were housed individually in clean cages with free access to food and water. Drugs were administered via gavage twice daily (200 µL per mouse), and the treatment was continued for 9 weeks. The control and model groups received saline via gavage at the same frequency and volume.

### CUBE 3D imaging

After isoflurane anesthesia, mice were positioned with their backs horizontally aligned. A rapid 3D skin imaging system (C-CUBE2, Pixience, France) was used to photograph the lower back skin. The camera lens was placed flush against the skin surface and oriented perpendicularly.

### OCT imaging

OCT (OSLF-1500, Shenzhen Certainn Technology Co., Ltd., China) was used to observe the vertical cross-section of the mouse back. The central wavelength was λ = 850 nm, and images were captured in line-scan mode with a resolution of 320 × 240 ppi. The lens height and orientation were adjusted until the image was clear and the signal was stable before image acquisition.

### HE staining

Full-thickness dorsal skin samples were collected from mice and fixed in 4% paraformaldehyde for 24 h. After dehydration, the samples were embedded in paraffin and sectioned into 6 μm slices. Following deparaffinization and rehydration, the sections were stained using an HE staining kit (G1120, Solarbio Science & Technology Co., Ltd., China). Gradient dehydration and xylene clearing were performed, and the sections were mounted with neutral resin. Images were captured using an inverted microscope (BX-51, Olympus Corporation, Japan). The epidermis and full-thickness skin were analyzed using ImageJ 8.0 software.

### Second-harmonic generation (SHG) imaging and MATLAB analysis

Isoflurane-anesthetized mice were positioned on the stage of a two-photon excited fluorescence (TPEF) microscope (FV1000MPE, Olympus Corporation, Japan). A holder was used to stabilize the dorsal skin, and SHG signals from the back were collected using a TPEF microscope equipped with a 25 × objective lens (numerical aperture 1.05). A Mai Tai DeepSee laser (100 femtoseconds, 80 MHz; MaiTai HP DS-OL, Spectra-Physics, California) was used with an excitation wavelength of 850 nm. Photoaged skin images were acquired from the epidermis to the dermis and stored as z-axis sequence images (z-axis step size: 2.5 μm). Images were captured at a resolution of 512 × 512 pixels with a scanning speed of 8 μm/pixel.

SHG images were converted to grayscale using MATLAB and denoised using Gaussian filtering to remove background noise. The orientation information of collagen fibers was extracted using the structure tensor function and visualized as pseudo-color images to represent spatial fiber orientation. Grayscale values were extracted from different image regions based on their grayscale distribution. Low grayscale values corresponding to hair follicle regions were excluded through threshold settings, and the total grayscale values were calculated to quantify collagen content.

### Masson’s trichrome staining

After deparaffinization and rehydration, skin tissue sections were stained using a Masson's Trichrome Staining Kit (G1340, Solarbio Science & Technology Co., Ltd., China). The sections were then dehydrated through an ethanol gradient, cleared in xylene, and mounted with neutral resin. Images were captured using an inverted microscope, and collagen fiber content was analyzed using ImageJ 8.0 software.

### Measurement of oxidative stress biomarkers

After isoflurane anesthesia, blood was collected from the retro-orbital venous plexus into 1.5 mL EP tubes. Samples were left at room temperature for 1 h, followed by overnight storage at 4 °C. Centrifugation was performed at 3000 r/min for 10 min, and the supernatant was transferred. SOD activity was measured using a SOD assay kit (A001-3, Nanjing Jiancheng Bioengineering Institute, China), and MDA content was determined using an MDA assay kit (A003-1, Nanjing Jiancheng Bioengineering Institute, China).

### Immunohistochemistry (IHC) staining

After deparaffinization and rehydration, antigen retrieval buffer (AR0026, Bioss Biotechnology Co., Ltd., China) was applied to skin tissue sections for 30 min. Following permeabilization and blocking, α-SMA-specific antibody (14395-1-AP, Proteintech Group Inc., 1:50) was added, and the sections were incubated overnight at 4 °C. After washing three times with PBS, sections were incubated with polymeric horseradish peroxidase (HRP)-labeled goat anti-mouse/rabbit IgG (PV-8000, Beijing Zhongshan Golden Bridge Biotechnology Co., Ltd., China) at room temperature for 1 h. Following another three washes with PBS, freshly prepared DAB or AEC chromogenic solution (PV-8000, Beijing Zhongshan Golden Bridge Biotechnology Co., Ltd., China) was added, and the sections were incubated at room temperature for 5 min. Finally, the sections were dehydrated through an ethanol gradient, cleared in xylene, mounted with neutral resin, and observed under an inverted microscope.

### Quantitative real-time polymerase chain reaction (qRT-PCR)

Total RNA was extracted from skin tissues and cell samples using trizol reagent (15596018, Invitrogen, USA).Complementary DNA (cDNA) was synthesized from 2 μg of total RNA using the ReverTra Ace qPCR RT Master Mix Kit (FSQ-201, TOYOBO Co., Ltd., Japan). qRT-PCR was performed using the SYBR Green Realtime PCR Master Mix Kit (QPK-201, TOYOBO Co., Ltd., Japan) on a PCR machine (1861096, Bio-Rad Laboratories, Inc., USA). Relative mRNA expression levels were determined using GAPDH as the reference gene and the relative standard curve method (2^(−ΔΔCt)^).

Primer sequences used in this study are listed in Supplementary Material 1.

### Western blotting (WB)

Total protein was extracted from skin tissues or cell samples, and its concentration was measured using a BCA Protein Assay Kit (P0012, Beyotime Biotech Inc., China).Total protein lysates (20–50 µg) were separated by SDS-PAGE. Specific proteins were detected using the following primary antibodies: Collagen I (14695-1-AP, Proteintech Group Inc., 1:1000), Collagen III (22734-1-AP, Proteintech Group Inc., 1:1000), TGFBR1 (30117-1-AP, Proteintech Group Inc., 1:1000), TGFBR2 (66636-1-Ig, Proteintech Group Inc., 1:1000), SMAD2 (12570-1-AP, Proteintech Group Inc., 1:1000), SMAD3 (66599-1-Ig, Proteintech Group Inc., 1:1000), SMAD2/3 (AP0548, ABclonal Biotechnology Co., Ltd., China, 1:1000), SMAD4 (10231-1-AP, Proteintech Group Inc., 1:1000), MMP1 (10371-2-AP, Proteintech Group Inc., 1:1000), TIMP1 (16644-1-AP, Proteintech Group Inc., 1:1000), MAPK14 (14064-1-AP, Proteintech Group Inc., 1:1000), JUN (24909-1-AP, Proteintech Group Inc., 1:1000), FOS (66590-1-Ig, Proteintech Group Inc., 1:1000), P16 (10883-1-AP, Proteintech Group Inc., 1:1000), P21 (10355-1-AP, Proteintech Group Inc., 1:1000), and P53 (10442-1-AP, Proteintech Group Inc., 1:1000), GAPDH (10494-1-AP, Proteintech Group Inc., 1: 1000),DYKDDDDK tag (66008-4-Ig, Proteintech Group Inc., 1:5000). Horseradish peroxidase-conjugated secondary antibodies (A0208/A0216, Beyotime Biotech Inc., China) and Clarity Western ECL Substrate (170-5061, Bio-Rad Laboratories, Inc., USA) were used to visualize target proteins. Images were captured using a chemiluminescence imaging system (MP ChemiDoc, Bio-Rad Laboratories, Inc., USA).

### Extraction of CCA lyophilized powder

A 10% CCA (Dong’e Ejiao, China) aqueous solution was prepared and placed into a 1 kDa dialysis bag. Distilled water was replaced every 4 h, and the process continued until the pH reached neutrality. The solution was centrifuged at 5000 r/min for 10 min to remove precipitates. The supernatant was collected and lyophilized using a freeze dryer (FD-1D-50, Boyikang Instrument Co., Ltd., China).

### Cells and cell culture conditions

BJ cells (human skin fibroblasts) were provided by Peking University Medical School. HaCaT cells (human keratinocyte cell line) and HDF cells (human dermal fibroblasts) were obtained from the National Biomedical Cell Resource Center. BJ and HaCaT cells were cultured in DMEM, while HDF cells were cultured in 1640 medium supplemented with 100 U/mL penicillin–streptomycin and 10% FBS (10099141, Invitrogen Corporation, USA). Cultures were maintained at 37 °C in a 5% CO₂ atmosphere. Cells were passaged twice using 0.25% (w/v) trypsin at a 1:2 ratio. Cell proliferation was measured using the Cell Counting Kit-8 (RM02823, ABclonal Biotechnology Co., Ltd., China).

### UV irradiation

Cells were cultured in six-well plates until 90% confluency. The medium was removed, and the cells were washed twice with PBS. A thin-layer PBS method was used for modeling. The cell culture plates were placed in the center of a UV box and irradiated under a UV lamp (SH4A, SIGMA, China) at a distance of 5 cm. For HaCaT cells, irradiation was performed four times within 48 h, with a total irradiation dose of 30–50 mJ/cm^2^. For BJ cells, irradiation was carried out five to six times over the course of one week, with a total irradiation dose ranging from 60.0 to 100 mJ/cm^2^. After each irradiation, cells were incubated in a 5% CO₂ atmosphere for a specified duration. Cells not exposed to UV irradiation served as untreated controls. After the final irradiation, the cells were incubated with 1000 ug/mL CCA lyophilized powder for either 24 or 48 h.

### Detection of intracellular ROS levels

Intracellular ROS levels were measured using the DCFH-DA assay (2,7-dichlorodihydrofluorescein diacetate).After removing the drug-containing medium, 10 μM DCFH-DA was added to each well. Cells were incubated at 37 °C for 20 min, washed three times with serum-free medium, and the fluorescence intensity was measured at excitation/emission wavelengths of 488/525 nm using a microplate reader. Fluorescence intensity was normalized to cell number.

### SA-β-gal assay

SA-β-gal activity was measured using the SA-β-gal staining kit (C0603, Beyotime Biotech Inc., China) according to the manufacturer’s instructions. Cells were fixed in fixation solution for 15 min, washed with PBS, and incubated with senescence detection solution at 37 °C. On the following day, blue-stained cells were imaged and counted under a microscope to determine the number of SA-β-gal-positive cells.

### Co-culture protocol for BJ and HaCaT cells

BJ cells were seeded into 6-well plates and cultured until reaching approximately 90% confluence. Cells were divided into three groups: Model, Model + Supernatant, and Model + Supernatant + CCA. The cell models were established according to the methods described in “UV irradiation” section. HaCaT cells were treated simultaneously on the last day of BJ cell modeling, receiving UV irradiation twice within 12 h, each lasting 20 min. The supernatant from HaCaT cells was collected after the first UV exposure and returned to the cells after irradiation. After the second UV exposure, the supernatant was again collected, thoroughly mixed, and immediately adjusted to a fixed volume. This mixed supernatant was then added to the modeled BJ cells. For the treatment group, 1000 µg/mL of lyophilized CCA powder was simultaneously added for 24 h.

### Peptide synthesis, purification, and stock solution preparation

The peptide was synthesized using solid-phase peptide synthesis (SPPS) and purified by high-performance liquid chromatography (HPLC), achieving a purity greater than 95%.FITC/FLAG tags were added at the N-terminus for detection and purification. HPLC analysis was performed using a SHIMADZU Inertsil ODS-SP column (4.6 × 250 mm, 5 µm). The mobile phase consisted of 0.1% trifluoroacetic acid (TFA) in water (Pump A) and 0.1% TFA in acetonitrile (Pump B), with a total flow rate of 1 mL/min. The purification process used a gradient elution method, with acetonitrile concentration gradually increasing and reaching 100% at 30 min, based on the peptide's characteristics.

The synthetic peptides had an initial mass of 10 mg. HFSVEGQLEFR (JT-181302, Synpeptide Co., Ltd, China) and YYTSASGDEMVSLK (JT-181303, Synpeptide Co., Ltd, China) was dissolved in 1 mL of 15% ACN + 85% H_2_O to prepare a 10 mg/mL stock solution. YYTSASGDEMVSLK-FITC (JT-186221, Synpeptide Co., Ltd, China) and YYTSASGDEMVSLK-FLAG (JT-186222**,** Synpeptide Co., Ltd, China) were dissolved in 1 mL of 20% ACN + 80% H_2_O to prepare 10 mg/mL stock solutions, stored at −20 °C. The appropriate volume of the stock solutions was diluted with serum-free medium to the desired concentrations, filtered for sterilization, and prepared fresh for immediate use.

### Immunofluorescence (IF) assay

Log-phase BJ cells were digested with trypsin and suspended in 10% FBS-DMEM at a density of 5 × 10^4^ cells/mL. A 100 μL cell suspension was seeded into each well of a 96-well fluorescence plate and incubated until adhesion. After removing the medium, cells were washed twice with PBS and incubated in 0.25 mg/mL YYTSASGDEMVSLK-FITC solution for 6 h in the dark. Cells were then washed three times with PBS, fixed with paraformaldehyde, permeabilized, and blocked. TGFBR1 and TGFBR2 antibodies were added for overnight incubation at 4 °C, followed by three PBS washes. Fluorescent secondary antibodies (AS054, ABclonal Technology, China) were added and incubated at 37 °C for 1 h in the dark. After washing with PBS, fluorescent dye containing DAPI (AB104139, Abcam, UK) was added. Images were captured using a super-resolution microscope (STEDYCON-20-VCLR-4-PH-PC-xx-FL-AFt, Abberior Instruments GmbH, Germany).

### Statistical analysis

Statistical analysis was performed using GraphPad Prism 8.0. The *Shapiro–Wilk* test was used to assess normality, with *P* > 0.1 indicating a normal distribution. Non-normal data were analyzed using the *Mann–Whitney* test. Homogeneity of variance was tested using the *F*-test or *Bartlett's* Test, with *P* > 0.1 indicating homogeneity. Normally distributed data with equal variance were analyzed using an unpaired *t*-test, while *Welch’s correction* was applied for unequal variances. Two-way or one-way *ANOVA* was used based on specific sample characteristics. Post hoc analysis of group differences was conducted using *Bonferroni* or *Tukey* tests. Data are presented as mean ± standard error of the mean (*SEM*), and *P* < 0.05 was considered statistically significant.

## Results

### Biological processes involving CCA peptides in mice

**Fig. 1:**
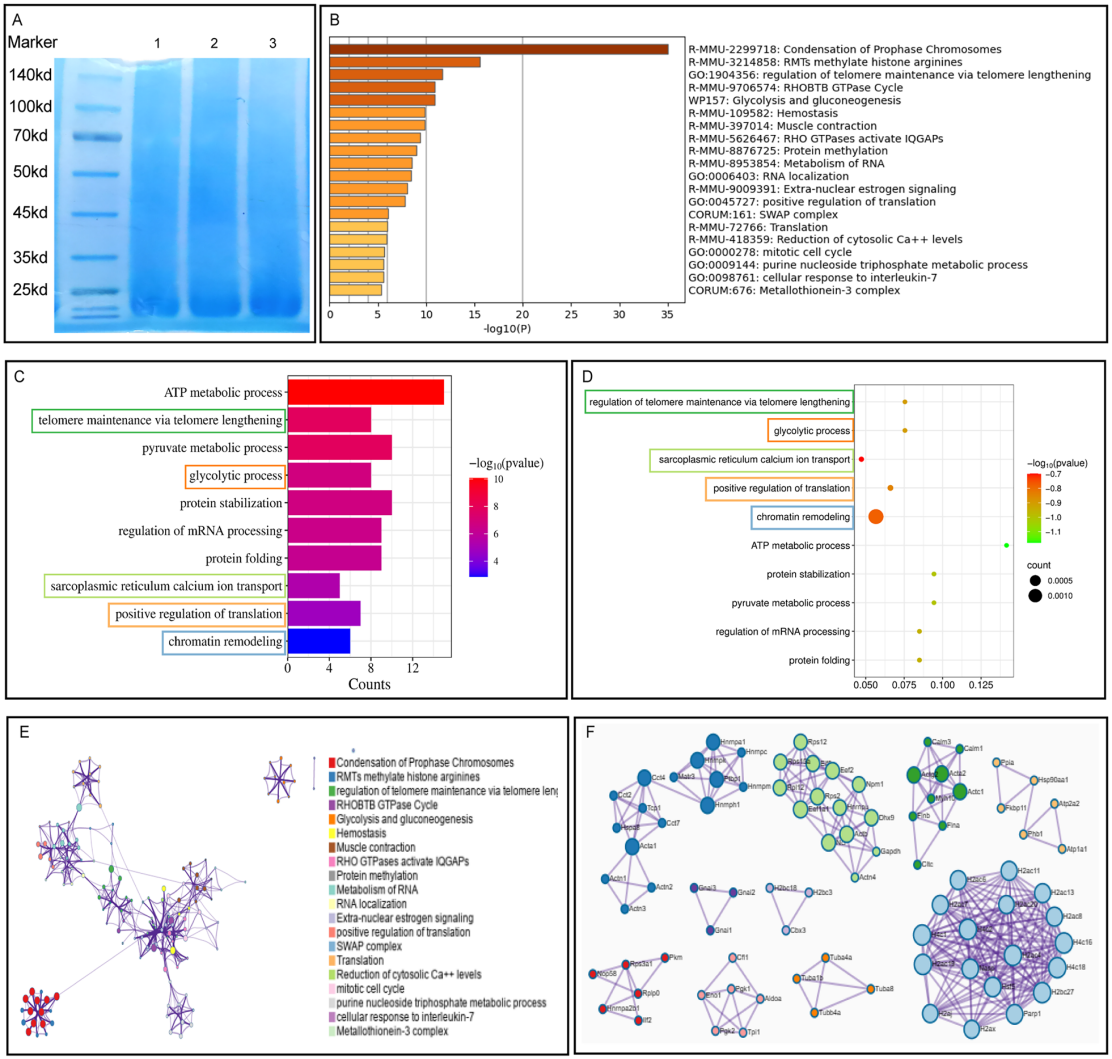
CCA peptides: involvement in mouse biological functions and PPI protein network. **A** SDS-PAGE analysis of CCA. **B–D** Characterization and functional prediction of CCA peptides. **B** Metascape enrichment analysis of CCA-derived peptides mapped to the mouse gene database. **C** Bar plot of Gene Ontology biological process enrichment analysis. **D** Bubble plot of GO biological process enrichment analysis (frame colors correspond to (**E**)). **E** PPI network. **F **Visualization of ten high-density MCODE modules

SDS-PAGE analysis of the CCA sample showed no distinct bands (Fig. 1A), suggesting that during the boiling process, macromolecules in CCA may have been converted into bioactive peptides. After sample purification and mass spectrometry identification, a total of 176 peptide fragments mapping to 98 proteins were identified, encompassing six major protein classes:molecular chaperones/heat-shock proteins (e.g., HSPD1, HSP90AA1); energy-metabolism enzymes (e.g., ENO1, PKM, GAPDH); cytoskeletal proteins (e.g., ACTB, ACTG1, TUBA/TUBB, MYH/MYL); translation-associated proteins (e.g., EEF1A1, EEF2); nuclear RNA-binding proteins (e.g., HNRNPA1, HNRNPK, HNRNPU); and histones (e.g., H2A, H2B, H3, H4). These proteins participate in key biological processes, including nuclear transcription, mRNA translation and transport, protein folding, and mitochondrial homeostasis. The peptides AFVHWYVGEGMEEGEFSEAR, AGLQFPVGR, and FDGALNVDLTEFQTNLVPYPR matched 19 proteins each, whereas AAVPSGASTGIYEALELR matched 15 proteins and HFSVEGQLEFR matched 12 proteins. These findings suggest that these peptides may represent key bioactive motifs through which CCA exerts its pharmacological effects. Notably, seven unique peptides were detected for HSPD1, indicating that this protein is either abundant or structurally stable in CCA and may play a pivotal role in its pharmacological activity (Supplementary Material 2).

The identified proteins were individually mapped to mouse gene databases using the Metascape platform for enrichment and PPI analyses. Metascape enrichment analysis indicated that CCA-derived peptides primarily participate in biological processes including condensation of prophase chromosomes, nucleosome assembly, and deposition of new CENPA-containing nucleosomes at centromeres. Additionally, these peptides were involved in carbohydrate metabolism, protein folding, and positive regulation of protein kinase and kinase activities (Fig. 1B). GO enrichment analysis revealed involvement in ATP metabolic processes, regulation of telomere maintenance via telomere lengthening, glycolysis, positive regulation of translation, and protein refolding, consistent with results from the Metascape analysis. We further employed the MCODE algorithm for PPI analysis of the 98 proteins, identifying 10 high-density modules. These modules were matched to enriched terms from Metascape and biological processes from GO analysis. Results showed that five high-density networks corresponded respectively to five biological processes: regulation of telomere maintenance via telomere lengthening, glycolytic process (glycolysis and gluconeogenesis), sarcoplasmic reticulum calcium ion transport (reduction of cytosolic Ca^2^⁺ levels), positive regulation of translation (translation), and chromatin remodeling (SWAP complex). Among these processes, positive regulation of translation and chromatin remodeling are closely associated with protein synthesis, while glycolysis provides energy required for protein synthesis. These findings suggest that bioactive peptides in CCA positively regulate biological processes in mice, aiding in genomic stability and regulating protein synthesis and its related functions.

### CCA improves the appearance of photoaged skin

**Fig. 2:**
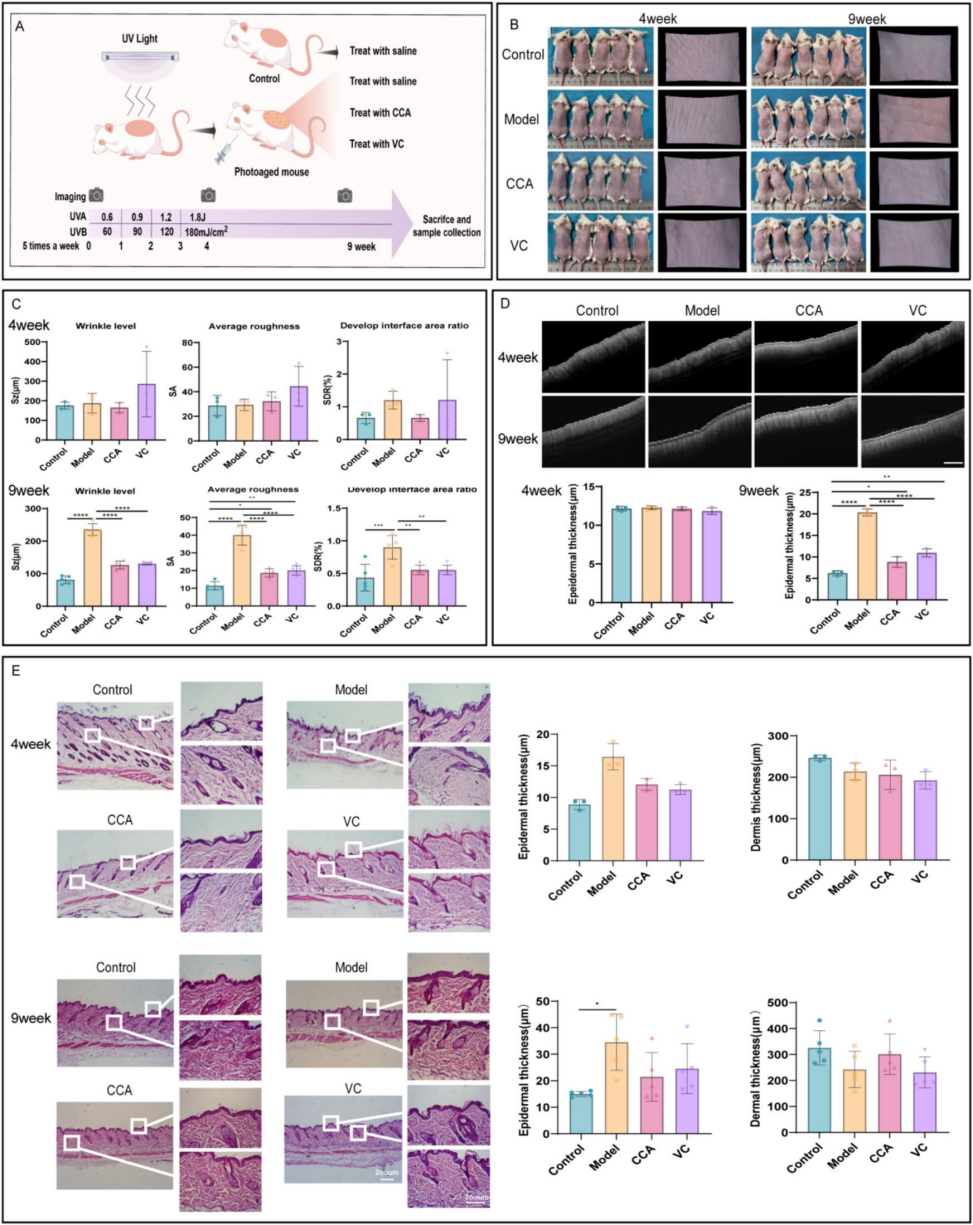
Effects of CCA on skin wrinkles and thickness in mice.** A **Schematic diagram of animal experiment (The Figure was generated using Figuredraw).** B, C** Imaging analysis of mouse skin appearance after 4 or 9 weeks of treatment with control, model, CCA, or VC (**B**). Images of mouse dorsal skin captured by camera and C-CUBE 3D imaging system at weeks 4 and 9 (**C**). Quantification of wrinkle levels and roughness of mouse dorsal skin at weeks 4 (n = 3) and 9 (n = 5). Scale bar = 2 mm. **D, E **Analysis of skin structure and thickness in mouse dorsal skin after 4 or 9 weeks of treatment with control, model, CCA, or VC (**D**). OCT imaging showing skin structure and quantification of epidermal thickness in mouse dorsal skin at weeks 4 and 9 (n = 3) (**E**). HE staining and statistical analysis of epidermal and dermal thickness in mouse dorsal skin at weeks 4 and 9, with insets and dashed lines indicating magnified regions of the epidermis and dermis at weeks 4 (n = 3) and 9 (n = 5). Scale bar = 200 µm. Data are presented as mean ± *SEM* (*P < 0.05, **P < 0.01, ***P < 0.001, ****P < 0.0001)

To evaluate the effect of CCA on improving photoaged dorsal skin in mice, changes in skin appearance were recorded using a camera and the C-CUBE 3D imaging system. At 4 weeks, the model group showed no significant changes in skin appearance. However, by 9 weeks, the model group exhibited significantly increased wrinkles and roughness, which were markedly improved following CCA intervention, with effects comparable to VC group (Fig. 2B, C). The specific effects of CCA on skin structure were further assessed using OCT in vivo imaging and HE staining. Results showed that epidermal thickness was significantly reduced following CCA treatment, with efficacy increasing in a time-dependent manner (Fig. 2D–E). These findings suggest that long-term oral administration of CCA can suppress UV-induced epidermal hyperplasia. At 4 weeks, dermal thickness in the CCA group showed no significant improvement. However, with prolonged treatment, dermal thickness in the CCA group gradually exceeded that of the model group, and by 9 weeks, there was no significant difference compared to the control group (Fig. 2E). Additionally, OCT signal intensity indicated that the dermal layer signal in the CCA group exceeded that of all other groups (Fig. 2D). Since the dermis primarily consists of collagen and elastic fibers, these findings suggest that long-term oral administration of CCA can enhance dermal collagen and elastic fiber content.

### CCA reduces dermal collagen damage in photoaged skin

**Fig. 3:**
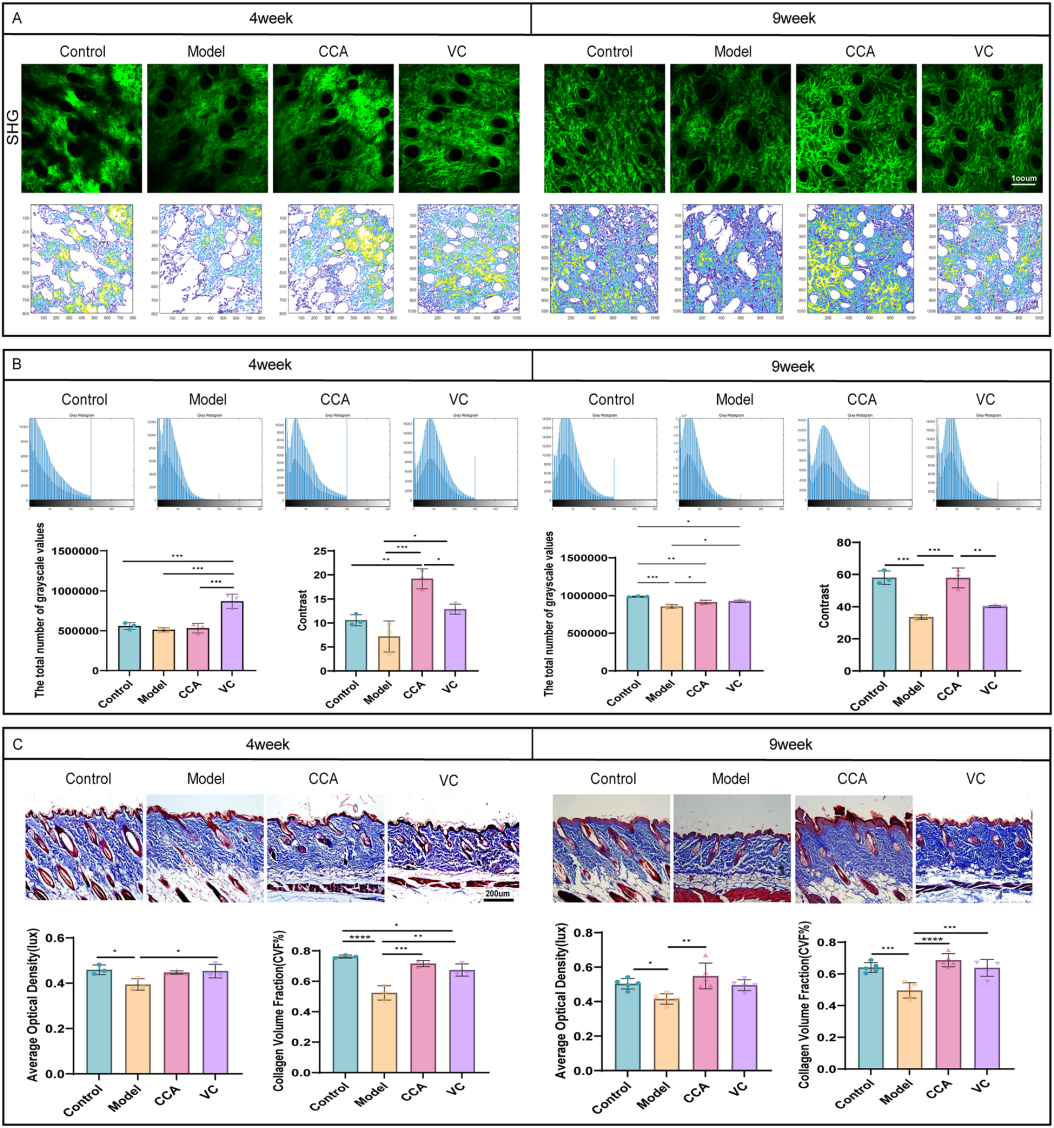
Effects of CCA on dermal collagen content in mice (**A, B**). Analysis of collagen structure in mouse dorsal skin using SHG imaging and MATLAB. (**A**). Top: SHG images of mouse dorsal skin at weeks 4 and 9 (scale bar = 100 µm). Bottom: Collagen texture structure maps extracted by MATLAB based on color depth (**B**). Top: Grayscale images of SHG processed by MATLAB, with gray intensity distribution maps after signal filling. Bottom: Statistical analysis of total grayscale values and contrast (n = 3). **C** Analysis of dermal collagen in mouse dorsal skin after 4 or 9 weeks of treatment with control, model, CCA, or VC. Masson staining of full-thickness dorsal skin sections from mice at weeks 4 and 9, with statistical analysis of dermal collagen volume fraction and density (week 4: n = 3, week 9: n = 5, scale bar = 200 µm). Data are presented as mean ± SEM (*P < 0.05, **P < 0.01, ***P < 0.001, ****P < 0.0001)

To investigate the effect of CCA on dermal collagen content in photoaged skin, second harmonic generation (SHG) imaging was used to visualize reticular collagen fibers in mouse dermis. Images were processed to grayscale, followed by signal filling to generate grayscale distribution maps. Results showed that after UV irradiation, the total grayscale value in the model group did not change significantly at week 4 but decreased significantly at week 9, indicating reduced continuity between collagen fibers. After CCA treatment, the grayscale distribution shifted significantly to the right at both time points, indicating pixel filling of dark regions. This suggests an increase in collagen fiber density and quantity, with the effect strengthening in a time-dependent manner (Fig. 3A, B). Texture analysis of the images using a grayscale co-occurrence matrix showed significant differences in contrast values between the CCA group and the control and model groups at week 4. This indicates that CCA improves collagen fiber morphology and stability in the early stages of photoaging, preventing excessive degradation. At week 9, contrast values in the CCA group increased significantly, exceeding those of the VC group. This indicates that collagen fibers in the CCA group were clearer and more structurally intact (Fig. 3B). MASSON staining further confirmed that at weeks 4 and 9, the model group exhibited loose collagen distribution, large gaps, and significant fiber breakage compared to the control group. In contrast, the CCA group showed substantial improvements in collagen fiber morphology and structure, with increased collagen density and quantity, which became more pronounced over time (Fig. 3C). In summary, long-term oral administration of CCA mitigates UV-induced collagen fiber damage and increases dermal collagen content in photoaged skin.

### CCA mitigates photoaging by reducing oxidative stress damage and inflammatory responses in photoaged skin while increasing collagen content

UV irradiation induces oxidative stress and inflammatory responses in the epidermis and dermis, with oxidative stress further exacerbating inflammation [26]. To investigate whether CCA can alleviate UV-induced oxidative stress and inflammation, we measured oxidative stress markers and inflammatory cytokine gene expression in the skin of photoaged mice.

**Fig. 4:**
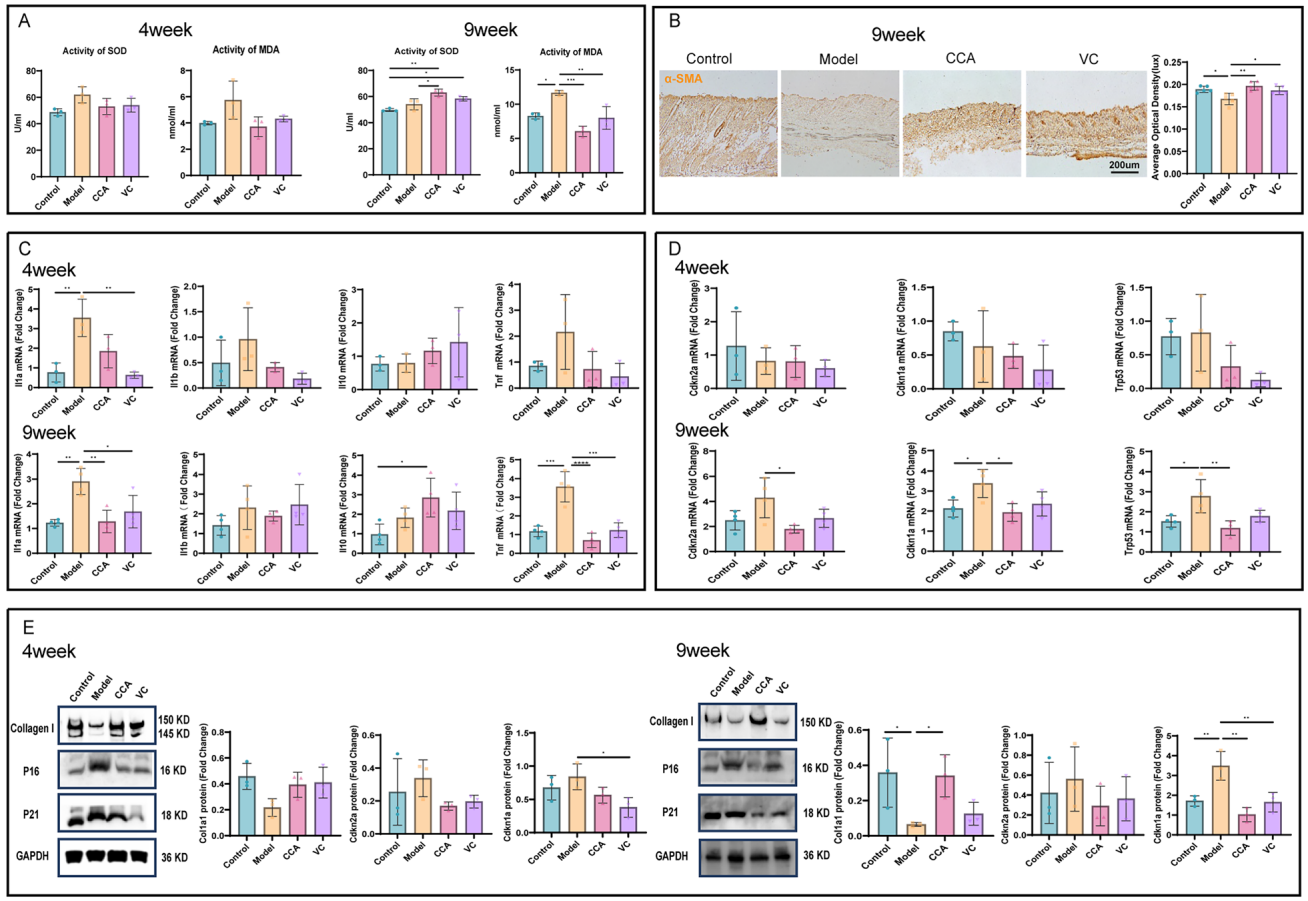
Effects of CCA on oxidative stress, inflammation, and aging markers in photoaged mouse dorsal skin. **A** Analysis of oxidative stress damage. measurement and statistical analysis of SOD and MDA levels in photoaged mouse dorsal skin treated with control, model, CCA, or VC for 4 or 9 weeks (n = 3). **B** α-SMA immunohistochemical staining and statistical analysis of mouse dorsal skin after 9 weeks of treatment with control, model, CCA, or VC (n = 5, scale bar = 200 µm). (*p < 0.05, **p < 0.01, *p < 0.001). **C, D** Gene expression analysis by RT-qPCR (**C**). RT-qPCR analysis of mRNA levels of inflammatory markers IL-1α, IL-1β, TNF-α, and IL-10 in mouse dorsal skin after 4 or 9 weeks of treatment with control, model, CCA, or VC (week 4: n = 3, week 9: n = 4) (**D**). RT-qPCR analysis of mRNA levels of aging markers P16, P21, and P53 in mouse dorsal skin after 4 or 9 weeks of treatment with control, model, CCA, or VC (week 4: n = 3, week 9: n = 4). **E** WB analysis and statistical evaluation of aging proteins and collagen I expression. WB results showing the expression levels of Collagen I, P16, and P21 in mouse dorsal skin after 4 or 9 weeks of treatment with control, model, CCA, or VC, with GAPDH used as a loading control (n = 3). Data are presented as mean ± *SEM* (*P < 0.05, **P < 0.01, ***P < 0.001, ****P < 0.0001)

The dynamic changes in SOD and MDA are important indicators of oxidative stress levels and cellular damage [27]. At week 4, the model group showed a rising trend in MDA levels, while SOD activity was higher than in the control and treatment groups, likely reflecting an early protective response to UV-induced stress aimed at maintaining homeostasis. Both the CCA and VC groups showed trends of mitigating oxidative stress. By week 9, the CCA group significantly increased SOD activity and suppressed MDA levels, demonstrating antioxidant effects that improved over time and exceeded VC group. These findings suggest that long-term oral administration of CCA can reduce oxidative stress damage. Furthermore, compared to week 4, the CCA group showed a trend of improvement over the control group at week 9 (Fig. 4A), suggesting that long-term oral administration of CCA may enhance the body's antioxidant capacity to prevent UV-induced oxidative stress damage.

RT-qPCR results showed that at week 4, both the CCA and VC groups exhibited trends of downregulating pro-inflammatory cytokine genes (IL-1α, IL-1β, TNF-α) and upregulating the anti-inflammatory cytokine IL-10, with the VC group outperforming the CCA group. At week 9, CCA significantly downregulated pro-inflammatory cytokines (IL-1α, IL-1β, TNF-α) and upregulated IL-10 expression. From week 4 to week 9, the CCA group progressively outperformed the VC group in reducing pro-inflammatory cytokine levels (Fig. 4C), suggesting that long-term oral administration of CCA alleviates UV-induced inflammation.

Collagen, synthesized and secreted by fibroblasts, plays a key role in maintaining skin structure and elasticity. Inflammation and oxidative stress reduce collagen content and quality, leading to skin aging, laxity, and wrinkle formation. At week 4 after UV irradiation, α-SMA staining was faint and showed no differences between groups. By week 9, α-SMA levels increased in the model group (Fig. 4B), indicating a transition of fibroblasts to the myofibroblast phenotype. Additionally, Collagen I expression decreased, while aging markers P16 and P21 increased at both gene and protein levels. The CCA group suppressed these changes, suggesting that long-term oral administration of CCA enhances collagen content and reduces aging marker expression, thereby mitigating photoaging. Compared to the VC group, the CCA group showed similar improvement at week 4 but demonstrated superior effects by week 9 (Fig. 4D, E).

### CCA regulates the dynamic balance between collagen synthesis and degradation via the TGFβ-SMAD/MMPs pathway

**Fig. 5:**
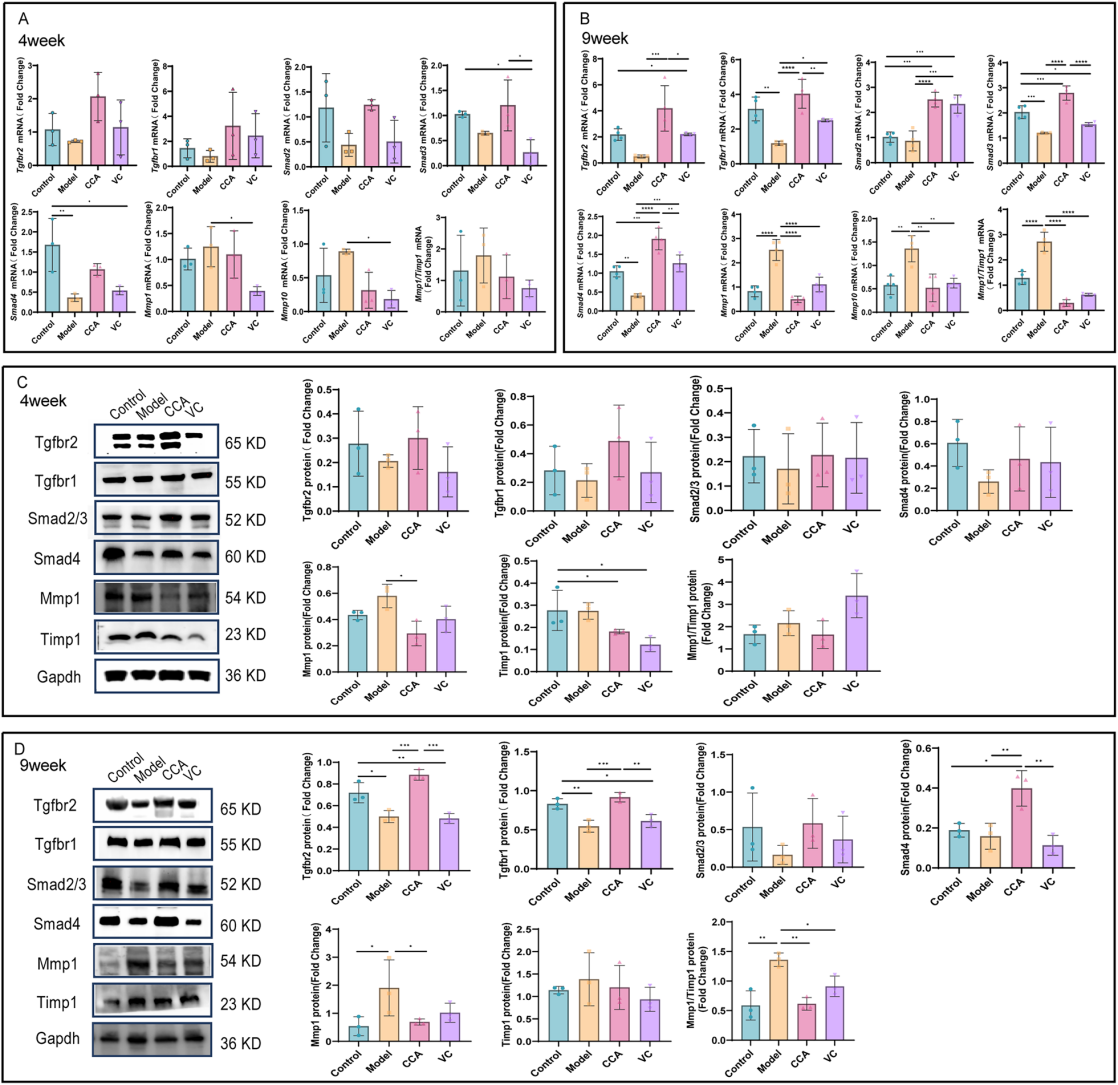
CCA regulates the dynamic balance of collagen synthesis and degradation via the TGFβ-SMAD/MMPs pathway. **A**, **B** RT-qPCR analysis and statistical evaluation of gene expression in the TGFβ-SMAD/MMPs pathway (**A**). RT-qPCR results showing mRNA expression levels of TGFBR2, TGFBR1, SMAD2, SMAD3, SMAD4, MMP1, and MMP10 in mouse skin after 4 weeks of treatment with control, model, CCA, or VC (n = 3) (**B**). RT-qPCR results showing mRNA expression levels of TGFBR2, TGFBR1, SMAD2, SMAD3, SMAD4, MMP1, and MMP10 in mouse skin after 9 weeks of treatment with control, model, CCA, or VC (n = 4). **C, D** WB analysis and statistical evaluation of protein expression in the TGFβ-SMAD/MMPs pathway (**C**). WB results showing protein expression levels of TGFBR2, TGFBR1, SMAD2/3, SMAD4, MMP1, and TIMP1 in mouse skin after 4 weeks of treatment with control, model, CCA, or VC, with GAPDH used as a loading control (n = 3) (**D**). WB results showing protein expression levels of TGFBR2, TGFBR1, SMAD2/3, SMAD4, MMP1, and TIMP1 in mouse skin after 9 weeks of treatment with control, model, CCA, or VC, with GAPDH used as a loading control (n = 3).Data are presented as mean ± *SEM* (*P < 0.05, **P < 0.01, ***P < 0.001, ****P < 0.0001)

The TGFβ-SMAD pathway plays a critical role in collagen synthesis.TGFBR1 activates SMAD2 and SMAD3, which specifically bind and form a phosphorylated SMAD2/3 complex. The phosphorylated SMAD2/3 complex associates with SMAD4 to form a biologically active transcription complex that translocates into the nucleus to regulate target gene transcription, thereby promoting Collagen I synthesis [28, 29]. After 4 weeks of UV exposure, the gene and protein expression levels of TGFBR1, TGFBR2, SMAD2, SMAD3, and SMAD4 in the model group showed a decreasing trend compared to the control group. By week 9, their expression was significantly reduced. At week 9, CCA significantly enhanced the synthesis of TGFBR1, TGFBR2, and SMAD4 at both gene and protein levels. It also increased the expression of SMAD2 and SMAD3, facilitating their assembly into the SMAD2/3 complex to some extent, this effect was time-dependent. Additionally, at week 4, the effects of CCA were comparable to those of the VC group. However, by week 9, CCA demonstrated significantly stronger upregulation of pathway proteins compared to VC, with expression levels nearly restored to those of the control group (Fig. 5A–D).

In photoaged skin, elevated oxidative stress factors activate NF-κB and MAPK-related pathway proteins, inducing the formation of the AP-1 transcription complex. This activation initiates the transcription and expression of the MMP family, increasing the MMPs/TIMPs ratio and leading to continuous collagen fiber degradation [30]. At both 4 and 9 weeks, the model group exhibited increased expression of MMP1 and MMP10 genes compared to the control group, showing a time-dependent increase. The MMP1/TIMP1 protein ratio also increased. CCA downregulated MMP1 gene and protein expression, reducing the MMP1/TIMP1 ratio. These results suggest that UV disrupts the dynamic balance between MMP1 and TIMP1, accelerating collagen degradation, whereas CCA inhibits collagen breakdown by restoring this balance. Moreover, at both time points, the MMP1/TIMP1 ratio in the CCA group was consistent with that of the control group and showed a trend superior to the VC group. This suggests that long-term oral administration of CCA may prevent collagen degradation (Fig. 5A–D).

### Protective effects of CCA against UV-induced epidermal and dermal cell damage

**Fig. 6:**
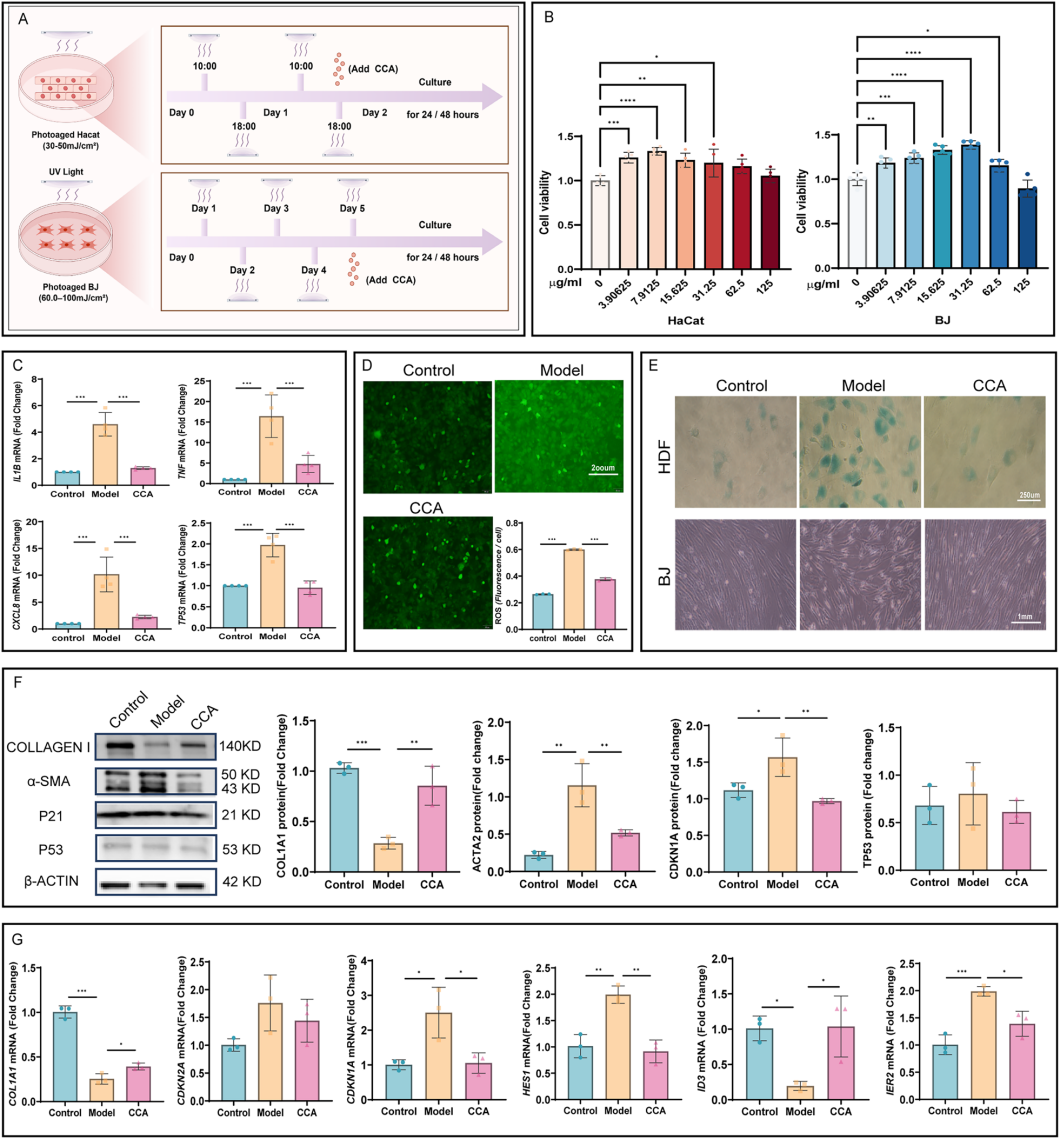
Changes in the aging phenotypes of keratinocytes and fibroblasts after CCA treatment.** A **Schematic diagram of cell experiment (The Figure was generated using Figuredraw). **B** Cell viability measured by CCK8 assay. CCA increases the viability of HaCaT and BJ cells. **C, D** Effects of CCA on inflammatory cytokines and ROS levels in HaCaT cells (**C**). RT-qPCR quantitative evaluation of mRNA expression levels of inflammatory cytokines IL-1β, IL-8, TNF-α, and aging marker P53 in HaCaT cells in control, model, and CCA groups (n = 3) (**D**). ROS fluorescence images and quantitative analysis of HaCaT cells in control, model, and CCA groups after CCA treatment (n = 3, scale bar = 200 µm). **E, F** Morphological changes and expression of aging markers in photoaged fibroblasts after CCA treatment (**E**). SA-β-gal staining of UV-irradiated HDF cells (scale bar = 250 µm) and images of BJ cell morphology (scale bar = 1 mm) (**F**). WB results showing the protein levels of Collagen I, α-SMA, P21, and P53 in BJ cells, along with quantitative analysis of protein expression, with β-actin used as the loading control (n = 3). **G** RT-qPCR analysis of mRNA expression levels of aging markers α-SMA, P16, P21, HES1, ID3, and IER2 in BJ cells in control, model, and CCA groups (n = 3). Data are presented as mean ± *SEM* (*P < 0.05, **P < 0.01, ***P < 0.001, ****P < 0.0001)

Literature indicates that UV irradiation affects different skin layers, damaging epidermal cell DNA and triggering the release of inflammatory cytokines, thereby inducing oxidative stress responses [31]. In the dermis, UV promotes the accumulation of ROS in fibroblasts, leading to collagen degradation and accelerated aging [32]. To further explore the differential regulatory effects of CCA on epidermal and fibroblast cells, in vitro experiments were conducted to separate skin structures (Fig. 6A). Cell viability after treatment was assessed using the CCK8 assay. The optimal CCA concentration was determined to be 7.9125 μg/mL for HaCaT cells and 31.25 μg/mL for BJ cells (Fig. 6B). Following UV exposure, HaCaT cells treated with the above CCA concentrations for 24 h exhibited significantly increased ROS levels, along with elevated expression of inflammatory cytokines (IL-1β, IL-8, TNF-α mRNA) and the aging marker P53 mRNA (Fig. 6C, D). This indicates exacerbated oxidative stress and inflammatory responses, accelerating cellular aging. CCA treatment for 24 h effectively alleviated UV-induced oxidative damage and cytokine secretion in epidermal cells. Prolonged UV exposure induced fibroblasts to transition to a senescent phenotype. HDF cells showed significantly increased SA-β-gal secretion, while UV-damaged BJ cells exhibited sparse arrangement and thinner morphology (Fig. 6E). α-SMA protein levels in fibroblasts increased significantly, suggesting a transition to the myofibroblast phenotype. Collagen I gene and protein levels were markedly reduced, while the expression of the aging markers P21 and P16 at the gene level and P21 and P53 at the protein level was upregulated. ID3 gene expression was significantly downregulated (Fig. 6F, G). However, unlike previous studies on natural aging [33], HES1 and IER2 gene expression levels increased rather than decreased, this discrepancy may result from differences in the mechanisms underlying natural aging versus photoaging. Nevertheless, after CCA treatment, the expression levels of these genes and proteins were consistent with those in the control group.

### CCA regulates fibroblast collagen synthesis and degradation via the TGFβ-SMAD/MMPs pathway

**Fig. 7:**
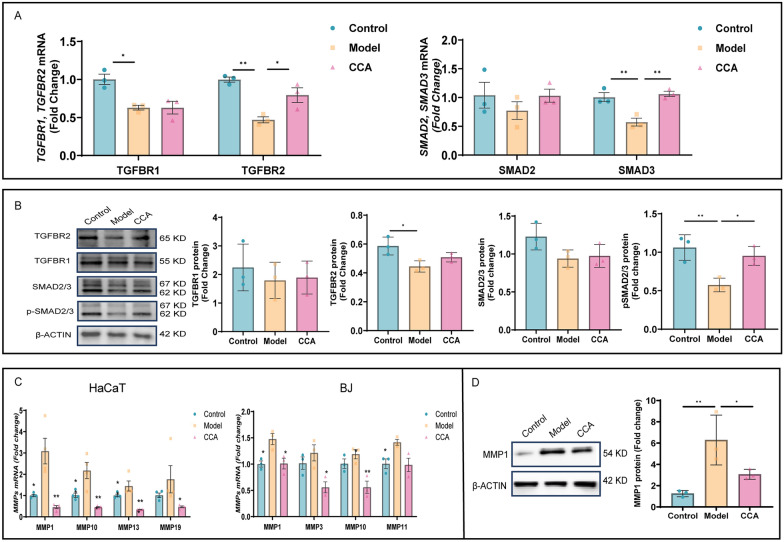
CCA regulates the expression of key molecules in the TGFβ-SMAD/MMPs pathway in BJ cells. **A** RT-qPCR gene expression analysis. RT-qPCR quantitatively evaluated mRNA expression levels of TGFBR1, TGFBR2, SMAD2, and SMAD3 in control, model, and CCA groups after CCA treatment (n = 3). **B** WB protein expression analysis. WB showed protein expression levels of TGFBR1, TGFBR2, SMAD2/3, and pSMAD2/3 in control, model, and CCA groups after CCA treatment, with GAPDH as the loading control (n = 3). **C, D** MMPs gene and protein expression analysis (**C**). RT-qPCR analysis of MMPs gene expression in HaCaT and BJ cells (n = 3) (**D**). Left: WB analysis showing MMP1 protein expression, with β-actin as the loading control. Right: quantitative analysis of MMP1 protein expression (n = 3). Data are presented as mean ± *SEM* (*P < 0.05, **P < 0.01, ***P < 0.001, ****P < 0.0001)

To investigate the potential mechanisms and targets of CCA in regulating fibroblast biological functions, we analyzed changes in mRNA and protein levels of TGFβ-SMAD pathway-related molecules in BJ cells. Results showed that CCA upregulated the mRNA and protein levels of TGFBR2, TGFBR1, SMAD2, and SMAD3 in BJ cells. It facilitated the binding of SMAD2/3 and significantly increased their phosphorylation levels, thereby enhancing Collagen I synthesis and secretion to stabilize skin structure and slow aging (Fig. 7A, B).

To identify the MMP family members expressed in fibroblasts, we analyzed the gene expression levels of 15 human MMPs. Compared to the control group, the expression of MMP1, MMP10, MMP13, and MMP19 was significantly elevated in HaCaT cells, while MMP1, MMP3, MMP10, and MMP11 were significantly upregulated in BJ cells. Among these, MMP1 exhibited the most pronounced changes. It is closely associated with aging and serves as the primary enzyme for degrading type I and type III collagen. Overexpression of MMP1 specifically degrades extracellular matrix components, disrupting the normal structure of collagen and elastic fibers, leading to wrinkles and other signs of aging.CCA inhibits collagen fiber degradation by reducing MMP1 protein levels, thereby maintaining skin elasticity (Fig. 7C, D).

### CCA inhibits the MAPK14 pathway to reduce interactions between photoaged epidermal and fibroblast cells

UVA primarily affects the dermis, while UVB predominantly targets the epidermis. During normal photoaging, both UVA and UVB simultaneously act on the skin, eliciting corresponding responses. Epidermal cells and fibroblasts are the two most critical cell types involved in photoaging. Literature indicates that collagen degradation is a key mechanism of photoaging, primarily driven by an imbalance in MMPs/TIMPs due to excessive MMPs secretion by dermal fibroblasts [34]. Although epidermal cells produce some MMPs through direct UV stimulation or autocrine mechanisms, their primary role in photoaging is mediating inflammatory responses. Intercellular communication plays a critical role in maintaining homeostasis and responding to environmental changes. Paracrine signaling is a key mechanism of intercellular communication, allowing inflammatory cytokines to exert their effects [35].

**Fig. 8:**
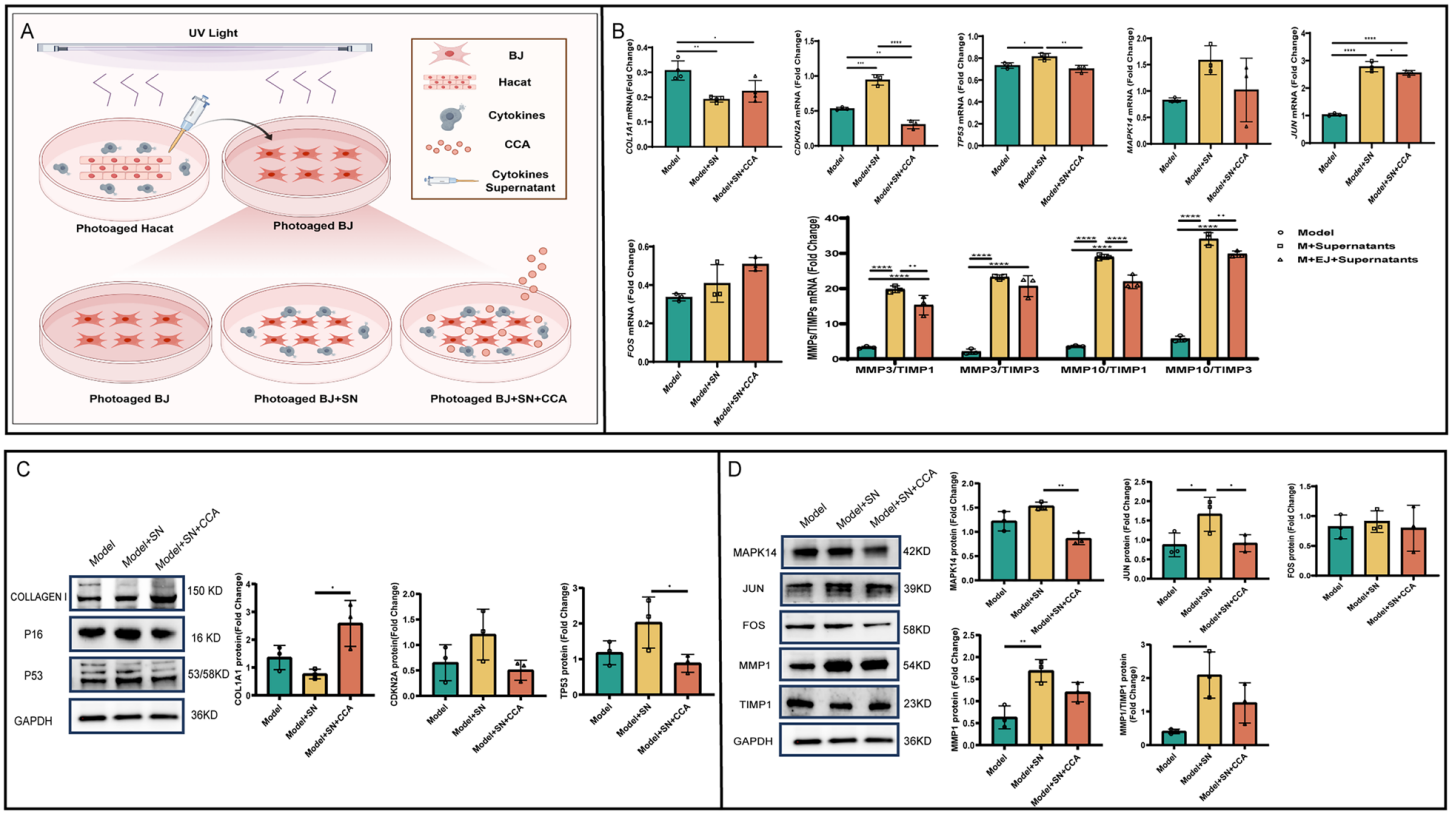
CCA inhibits the paracrine effects of epidermal cells on fibroblasts via the MAPK14 pathway. **A **Schematic diagram experiment (The Figure was generated using Figuredraw). **B** RT-qPCR gene expression analysis. RT-qPCR quantitatively evaluated mRNA expression levels of Collagen I, P16, P53, MAPK14, FOS, JUN, and MMPs/TIMPs in model, model + SN, and model + SN + CCA groups after CCA treatment (n = 6). **C, D** WB protein expression analysis (**C**). WB analysis showing protein expression levels of Collagen I, P16, and P53, with GAPDH used as the loading control (n = 3). Quantitative analysis of protein expression is included (**D**). WB analysis showing protein expression levels of MAPK14, FOS, JUN, MMPs, and TIMPs, with GAPDH used as the loading control (n = 3). Quantitative analysis of protein expression is included. Data are presented as mean ± *SEM* (*P < 0.05, **P < 0.01, ***P < 0.001, ****P < 0.0001)

To investigate whether epidermal inflammatory cytokines influence the MMPs/TIMPs ratio in fibroblasts via paracrine signaling, accelerating dermal collagen degradation, and whether CCA can inhibit this effect, UV-irradiated HaCaT supernatants (SN) were co-cultured with BJ cells (Fig. 8A). Relevant molecular changes were assessed using PCR and WB. Results indicated that, compared to the model group, the model + SN group exhibited a significant increase in the MMPs/TIMPs ratio, along with upregulated expression of aging markers P16 and P53 at both gene and protein levels, and decreased Collagen I expression. These findings suggest that inflammatory cytokines secreted by epidermal cells act on fibroblasts via paracrine signaling, accelerating collagen degradation and cellular aging. Notably, CCA was able to reverse these effects (Fig. 8B, C). Studies indicate that inflammatory cytokines activate the MAPK14 pathway, promoting the formation of the AP-1 complex by JUN and FOS. This initiates high MMPs expression and increases the MMPs/TIMPs ratio [36]. To determine whether CCA reduces MMPs expression and the MMPs/TIMPs ratio by inhibiting the MAPK14 pathway in fibroblasts, thereby mitigating collagen degradation and aging, MAPK14 pathway molecules were analyzed using PCR and WB. Results showed that CCA intervention significantly downregulated the expression of MAPK14 and JUN proteins in photoaged BJ cells, with minimal effect on FOS, suggesting that MAPK14 and JUN may serve as core targets for the effects of CCA. Furthermore, CCA can reduced the MMPs/TIMPs ratio to some extent. These findings suggest that CCA may mitigate photoaging by inhibiting the MAPK14 signaling pathway in fibroblasts, mediated by epidermal inflammatory cytokines via paracrine signaling (Fig. 8B–D).

### Pharmacodynamic validation of core CCA peptides HFSVEGQLEFR and YYTSASGDEMVSLK

**Fig. 9:**
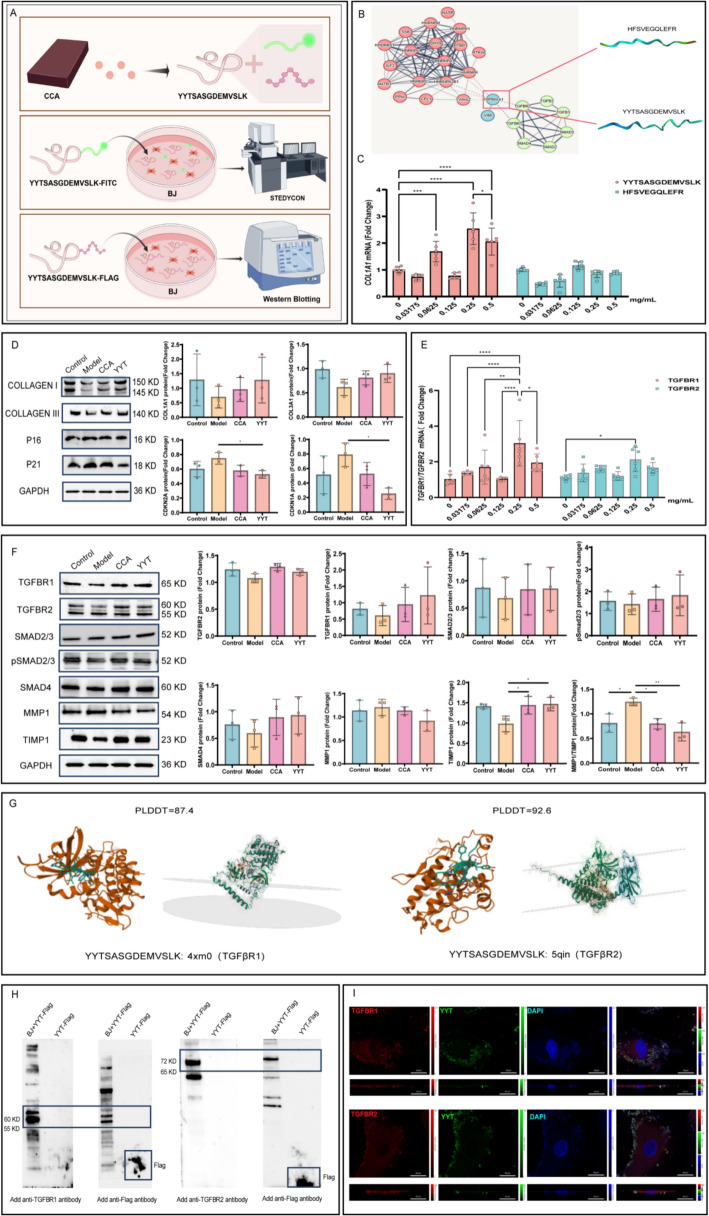
Pharmacological effects and colocalization analysis of CCA peptides. **A **Schematic diagram of peptide experiment (The Figure was generated using Figuredraw). **B** PPI analysis of CCA peptides with the TGFβ-SMAD pathway and visualization of AlphaFold3-predicted structures. Left: PPI analysis of CCA peptides with the TGFβ-SMAD pathway. Right: AlphaFold3-predicted structures of YYTSASGDEMVSLK and HFSVEGQLEFR. **C, D** Pharmacological validation of peptides (**C**). RT-qPCR analysis of Collagen I mRNA expression levels after treatment with different concentrations of YYTSASGDEMVSLK and HFSVEGQLEFR (n = 6) (**D**). WB analysis showing protein expression levels of P21, P16, Collagen I, and Collagen III in different groups, with GAPDH used as the loading control (n = 3). **E, F** Regulation of TGFBR1 and TGFBR2 gene and protein expression by YYTSASGDEMVSLK to activate the TGFβ-SMAD pathway (**E**). RT-qPCR analysis of TGFBR1 and TGFBR2 mRNA expression levels in photoaged BJ cells treated with 0.25 mg/mL YYTSASGDEMVSLK (n = 6) (**F**). WB analysis showing protein expression levels of TGFBR2, TGFBR1, SMAD2/3, pSMAD2/3, SMAD4, MMP1, and TIMP1 in different groups, with GAPDH used as the loading control (n = 3). **G** Visualization of AlphaFold3-predicted interactions between YYTSASGDEMVSLK and TGFBR1/TGFBR2. orange: AlphaFold3-predicted PLDDT values and 3D structures of YYTSASGDEMVSLK bound to TGFBR1 (PDB ID: 4xm0) and TGFBR2 (PDB ID: 5qin). green: schematic representation of colocalization of YYTSASGDEMVSLK with TGFBR1 and TGFBR2 on the cell membrane. **H, I** Colocalization analysis of YYTSASGDEMVSLK with TGFBR1 and TGFBR2 (**H**). WB analysis showing binding of YYTSASGDEMVSLK-Flag to TGFBR1 and TGFBR2 in BJ cells incubated with 0.25 mg/mL YYTSASGDEMVSLK-Flag for 24 h (**I**). STEDYCON imaging showing colocalization of YYTSASGDEMVSLK-FITC (green) with TGFBR1 and TGFBR2 (red). Yellow indicates colocalization, and nuclei are stained with DAPI (blue) (scale bar = 20 µm). Data are presented s mean ± *SEM* (*P < 0.05, **P < 0.01, ***P < 0.001, ****P < 0.0001)

To further investigate the pharmacological basis of CCA (Fig. 9A), PPI analysis was performed between the CCA-derived proteins and TGFβ-SMAD pathway proteins. Results indicated that HSP90AA1 protein in CCA interacts with TGFBR1/TGFBR2 (Fig. 9A, B). Mass spectrometry revealed that this protein contains two peptide sequences: HFSVEGQLEFR and YYTSASGDEMVSLK (Supplementary Material 2). Sequence comparison further showed that both human and mouse HSP90AA1 proteins include the complete peptide sequences HFSVEGQLEFR and YYTSASGDEMVSLK (Supplementary Material 3), Therefore, we synthesized these two peptides and verified their biological activity through cell assays. To further validate and identify the specific small peptide responsible for the pharmacological effects of CCA, RT-qPCR was used to assess the expression levels of Collagen I, TGFBR1, and TGFBR2 genes in photoaged BJ cells 24 h after treatment. The results indicated that 0.0625, 0.25, and 0.5 mg/mL YYTSASGDEMVSLK significantly increased Collagen I transcription levels, with 0.25 mg/mL YYTSASGDEMVSLK showing the most pronounced upregulation of Collagen I gene expression. In contrast, different concentrations of HFSVEGQLEFR showed little or even inhibitory effects on Collagen I gene regulation in photoaged BJ cells (Fig. 9C).

Therefore, YYTSASGDEMVSLK was further selected to evaluate its effects on collagen synthesis and the expression of aging-related proteins in photoaged BJ cells using WB. The results showed that YYTSASGDEMVSLK promoted the expression of type I and type III collagen while significantly reducing the protein levels of aging markers P16 and P21. Notably, this peptide exhibited a trend of superiority over CCA (Fig. 9D). To further explore whether YYTSASGDEMVSLK promotes collagen synthesis and inhibits collagen degradation by activating the TGFβ-SMAD/MMPs pathway, PCR and WB were performed to assess the expression of related molecules. Considering that the optimal concentration of YYTSASGDEMVSLK may differ for TGFBR1 and TGFBR2, screening for the core targets was combined with further evaluation of the optimal concentration of YYTSASGDEMVSLK acting on TGFBR1 and TGFBR2. The results indicated that except for 0.125 mg/mL, other concentrations of YYTSASGDEMVSLK upregulated the gene expression of TGFBR1 and TGFBR2, with 0.25 mg/mL significantly enhancing the transcription level of TGFBR1, which was superior to TGFBR2, this concentration aligned with the optimal concentration for promoting Collagen I transcription. WB results further confirmed that YYTSASGDEMVSLK increased the protein expression of TGFBR1 and TGFBR2, with TGFBR1 showing a trend of higher expression than TGFBR2, consistent with the PCR results. Additionally, this peptide upregulated the protein levels of SMAD2/3, pSMAD2/3, and SMAD4 in BJ cells, comparable to the effects of CCA. Furthermore, YYTSASGDEMVSLK significantly increased TIMP1 expression and decreased the MMP1/TIMP1 ratio, thereby inhibiting collagen degradation. These findings further clarify the mechanism of action of YYTSASGDEMVSLK (Fig. 9E, F).

To investigate whether YYTSASGDEMVSLK binds to TGFBR1/TGFBR2 to initiate collagen synthesis, the three-dimensional structure of YYTSASGDEMVSLK was first predicted using the AlphaFold3 platform, and its PLDDT values for binding to TGFBR1/TGFBR2 were evaluated. Higher PLDDT values indicate greater accuracy of structural predictions in the corresponding region. The results showed that the PLDDT value for YYTSASGDEMVSLK binding to TGFBR1 (PDB ID: 4xm0) was 87.4, while the PLDDT value for binding to TGFBR2 (PDB ID: 5qin) was 92.6, indicating high confidence in the predicted interaction regions between YYTSASGDEMVSLK and TGFBR1/TGFBR2, and suggesting reliable results (Fig. 9G). Next, to verify the accuracy of these predictions, YYTSASGDEMVSLK was labeled with both FITC and FLAG molecules and applied to photoaged BJ cells. WB and IF were used to examine the colocalization of the peptide with its targets. WB results demonstrated overlapping bands after sequential incubation with TGFBR1/TGFBR2 and FLAG antibodies, with the protein molecular weights larger than those of the original TGFBR1 and TGFBR2 (Fig. 9H), indicating that YYTSASGDEMVSLK-FLAG binds to TGFBR1 and TGFBR2. Super-resolution IF results showed that YYTSASGDEMVSLK-FITC colocalized with both TGFBR1 and TGFBR2, with the yellow fluorescence representing colocalization with TGFBR1 being more prominent than that with TGFBR2 (Fig. 9I), suggesting that the core CCA peptide YYTSASGDEMVSLK has a higher binding affinity for TGFBR1 compared to TGFBR2.

## Discussion

Photoaging is the result of prolonged exposure to both UVA and UVB, leading to a dynamic imbalance between collagen synthesis and degradation. UVB primarily affects the epidermis, causing acute damage, while UVA penetrates into the dermis, directly disrupting collagen structure and causing fragmentation, disorganization, or cross-linking [37]. Simultaneously, UV induces excessive ROS production and inflammatory responses in skin cells, which not only oxidize collagen, leading to fiber degeneration, but also activate key signaling pathways such as TGFβ-SMAD/MMPs, further inhibiting collagen synthesis and accelerating degradation. The severity of photoaging has become a global public health issue, particularly with the aging population and increased sun exposure, significantly escalating its socioeconomic burden. The balance between collagen synthesis and degradation is considered a central regulatory mechanism in photoaging, making the promotion of collagen regeneration a key research focus in addressing photoaged skin.

According to Compendium of Materia Medica, CCA is traditionally used orally as a tonic. Historical documents and modern clinical practices have consistently adopted oral administration. In this study, we continued this traditional administration method to better simulate real clinical conditions and accurately evaluate its efficacy. In contrast, modern pharmacological studies have demonstrated that orally administered collagen can be transformed into more bioactive small molecules. These molecules can continuously deliver active peptides to the dermis via blood circulation, directly targeting lesion sites [16, 38]. In recent years, polyphenols and polysaccharides derived from natural plants and fungi have attracted significant attention for their potential to prevent and alleviate UV-induced skin photodamage. Hasegawa et al. reported that the tea polyphenol chafuroside B inhibits RANKL gene expression in keratinocytes, reducing levels of inflammatory mediators TNF-α, PGE_2_, and IL-10 [39]. Potapovich et al. demonstrated that plant polyphenols such as resveratrol, quercetin, and verbascoside regulate inflammatory responses in keratinocytes via NF-κB, aryl hydrocarbon receptor, and EGFR-ERK signaling pathways. Among these, the NF-κB pathway is considered essential for inflammation regulation [40]. Lycium barbarum polysaccharide, a natural polysaccharide, exerts antioxidant effects via the SIRT3-SOD2 pathway, alleviating UVB-induced skin cell aging [41]. Du et al. also confirmed that β-glucans derived from algae, cereals, and shiitake mushrooms enhance the production of antioxidant enzymes such as SOD and MDA. This activity improves clearance of ROS including superoxide anions and hydroxyl radicals, thus preventing UV-induced oxidative stress and delaying skin aging [42]. These studies suggest that reported polyphenols and polysaccharides mainly mitigate photoaging via anti-inflammatory and antioxidant pathways. Although inflammation and oxidative stress are critical early pathological factors in photoaging, the ultimate driver of skin aging is the irreversible degradation of collagen structure and function. Thus, merely eliminating ROS or inflammatory factors without restoring collagen homeostasis provides limited improvement to aging skin phenotypes. Conversely, supplementing collagen or inhibiting MMP activity without addressing persistent oxidative stress fails to maintain long-term ECM integrity. In summary, a comprehensive and effective anti-photoaging strategy should integrate antioxidant, anti-inflammatory, and collagen homeostasis restoration functions to address the entire pathological process of photoaging. CCA has a complex composition and exerts pharmacological effects through multi-target and multi-pathway mechanisms. It not only possesses anti-inflammatory and antioxidant properties but also supplements dermal collagen or promotes fibroblast collagen secretion [43], this study further demonstrated that CCA reduces collagen degradation by inhibiting inflammation- and ROS-induced MMPs production. Additionally, it promotes collagen synthesis and improves collagen structure via activation of the TGFβ-SMAD signaling pathway, providing comprehensive, multi-pathway protection against photoaging.

After digestion and absorption in the gastrointestinal tract, active components of collagen peptides circulate through the bloodstream, targeting skin cells and exerting protective effects. Watanabe-Kamiyama et al. administered ^14^C-labeled low-molecular-weight collagen hydrolysate orally to rats, demonstrating a rapid increase in plasma radioactivity. Fibroblasts were shown to uptake intact Gly-^14^C-Pro-Hyp or ^14^C-Pro-Hyp peptides, accumulating these peptides in the skin at elevated levels for up to 14 days [38]. Iwai et al. conducted human trials and confirmed that oral intake of low-molecular-weight collagen hydrolysates significantly elevated Hyp-containing peptide levels in the blood, peaking within 1–2 h post-ingestion [44]. Shigemura et al. further utilized LC–MS/MS analysis to demonstrate that various Hyp-related dipeptides and tripeptides could be simultaneously detected in human plasma following ingestion of collagen hydrolysates [45]. Studies have shown that small peptide fragments exhibit higher bioactivity and safety. Collagen peptides have been demonstrated to possess various physiological activities, supplementing collagen or promoting its synthesis to improve photoaged skin [14, 16, 46]. Therefore, identifying active peptides capable of activating the body’s self-repair mechanisms is crucial for improving photoaging. Mass spectrometry analysis has revealed that CCA is rich in small peptides, highlighting its great potential for development as a drug for delaying photoaging. However, current research primarily focuses on the ameliorative effects of CCA on UV-induced photoaging phenotypes in animal and cellular models, with limited exploration of its active components and precise mechanisms. This study, for the first time, investigates the core targets and mechanisms of CCA and its key small peptide fragments in mitigating photoaging through the dynamic processes of collagen synthesis and degradation.

To investigate the biological processes and pathways potentially involved in CCA, we performed enrichment analysis and PPI analysis using the corresponding proteins of the 98 CCA peptides identified by mass spectrometry. The analysis revealed that CCA peptides are primarily involved in processes such as condensation of prophase chromosomes and RMTS methylation of histone arginines, both of which contribute to the stability of genetic material. Additionally, we focused on the pathways of positive regulation of translation and translation, which regulate and execute the translation of mRNA, collectively promoting protein synthesis. The SWAP complex and RNA localization pathways influence RNA processing, transport, and localization, ensuring the correct positioning of mRNA within the cell. These mechanisms enhance translation efficiency and optimize protein synthesis, providing a basis for CCA’s regulation of collagen synthesis.

This study established a photoaging mouse model and used advanced optical imaging combined with traditional ex vivo experiments to comprehensively observe changes in different skin structures of the dorsal region at weeks 4 and 9. Inflammatory factors, oxidative stress markers, and aging indicators were assessed to evaluate the pharmacological effects of CCA in alleviating photoaging. Results showed that the CCA group effectively reduced skin wrinkles and roughness, decreased epidermal thickness, inhibited the transformation of fibroblasts into myofibroblasts, and increased dermal collagen fiber content. At both 4 and 9 weeks, the CCA group significantly reduced the levels of pro-inflammatory factors IL-1α, IL-1β, and TNF-α, while increasing the expression of the anti-inflammatory factor IL-10. Furthermore, compared to the model group, the CCA group decreased SOD activity and increased MDA levels, thereby mitigating oxidative stress damage. These effects exhibited a time-dependent enhancement. Pharmacological validation suggests that long-term oral administration of CCA reduces the expression of aging markers by increasing collagen content and decreasing inflammation and oxidative stress responses, thereby alleviating photoaging. In addition, we further validated the effects of CCA on the morphology and function of epidermal and fibroblast cells through in vitro experiments. Results demonstrated that CCA improved cell viability, enhanced cell morphology, and alleviated inflammation and oxidative stress damage, thereby increasing type I collagen expression and reducing the senescence-associated secretory phenotype.

In our study, we observed significant changes in inflammatory markers both in vitro and in vivo. Skin is an essential immune organ containing various immune cells, among which macrophages play a critical role in the inflammatory response triggered by UV exposure. Classically activated macrophages (M1) promote acute and chronic inflammation induced by UV exposure through the production of pro-inflammatory mediators, recruitment of immune cells, and release of MMPs and ROS that degrade dermal ECM [47, 48]. Alternatively activated macrophages (M2) maintain skin homeostasis by upregulating anti-inflammatory factors and clearing tissue debris [49]. Our results showed that CCA significantly reduced pro-inflammatory cytokines and increased anti-inflammatory cytokines in photoaged mice in a time-dependent manner. Epidermal cells are primary mediators of UV-induced inflammation, and our cell experiments also demonstrated that CCA markedly decreased pro-inflammatory cytokines in HaCaT cells. These findings suggest that CCA might alleviate inflammation by modulating the M1/M2 macrophage ratio—specifically, by inhibiting M1 activation and reducing pro-inflammatory cytokine release while promoting M2 activation and enhancing anti-inflammatory cytokine production. After UV-induced acute skin inflammation subsides, a prolonged immunosuppressive resolution phase occurs concurrently with tissue repair processes [50]. During this phase, IL-10-positive neutrophils and macrophages significantly infiltrate human skin [51, 52]. If UV exposure ceases, immunosuppression gradually resolves as tissue repair completes, and skin immunity returns to normal levels. However, prolonged UV exposure leads to persistent chronic immunosuppression, limiting inflammatory responses while exacerbating ECM aging and skin immune dysfunction. Animal experiments indicated that after 9 weeks, expression of pro-inflammatory cytokines (IL1A, IL1B, TNF) in the CCA group decreased to levels comparable to the control group, suggesting that CCA has moderate regulatory effects, preventing excessive suppression of inflammation and maintaining essential skin immune defenses. Whether long-term CCA regulation of IL-10 expression gradually approaches normal levels requires further extended observation.

In fibroblasts, collagen synthesis and degradation are dynamically balanced to maintain extracellular matrix stability. When collagen synthesis decreases or degradation becomes excessive, skin elasticity declines, accelerating the aging process. The TGFβ-SMAD pathway plays a critical role in regulating collagen synthesis. Both animal and cell experiments demonstrated that CCA upregulates the gene and protein expression of TGFBR1, TGFBR2, SMAD2, SMAD3, and SMAD4, promoting SMAD2 and SMAD3 binding and phosphorylation, thereby activating the TGFβ-SMAD pathway and increasing type I collagen levels. The activation and upregulation of MMP family genes accelerate collagen degradation, whereas the TIMP family counteracts this effect, Thus, the dynamic balance between MMPs and TIMPs is crucial for regulating collagen degradation. Animal experiments showed that CCA reduces MMP1 and MMP10 mRNA expression and decreases the MMP1/TIMP1 gene ratio. WB further confirmed that CCA treatment reduced MMP1 protein levels and the MMP1/TIMP1 ratio in mouse skin. Cellular experiments also demonstrated that CCA significantly downregulated the mRNA expression of multiple MMPs in photoaged HaCaT and BJ cells, thereby reducing collagen degradation. In summary, CCA likely promotes collagen synthesis by activating the TGFβ-SMAD pathway and regulates the dynamic balance of MMPs/TIMPs to inhibit excessive collagen degradation, effectively reducing the secretion of aging markers P21 and P16, thereby ameliorating photoaging phenotypes. Literature indicates that UV-induced inflammatory factors secreted by epidermal cells act on dermal fibroblasts through paracrine signaling, activating the MAPK14 pathway in fibroblasts, accelerating AP-1 transcription complex formation, increasing MMPs expression, and disrupting the MMPs/TIMPs ratio, further accelerating collagen degradation. Thus, this intercellular crosstalk exacerbates photoaging. Our results showed that the inflammatory supernatant from UV-irradiated HaCaT cells, when applied to photoaged BJ cells, accelerated Collagen I degradation and increased the accumulation of aging markers P16 and P53, exacerbating photoaging, consistent with previous findings [53]. CCA alleviates photoaging by inhibiting the mRNA and protein expression of MAPK14, JUN, and FOS in the MAPK14 pathway, subsequently downregulating MMP1 expression, reducing the MMP1/TIMP1 ratio, and mitigating collagen degradation and aging marker secretion.

To identify the key components of CCA responsible for its pharmacological effects, PPI analysis predicted that the core peptides YYTSASGDEMVSLK and HFSVEGQLEFR interact with the TGFβ-SMAD pathway, targeting TGFBR1 and TGFBR2. RT-qPCR analysis of type I collagen gene expression confirmed the significant pharmacological activity of YYTSASGDEMVSLK. WB further demonstrated that YYTSASGDEMVSLK increased type I and III collagen protein levels while significantly reducing aging proteins P16 and P21, outperforming the CCA group. Subsequently, AlphaFold3 was used to predict the interaction between YYTSASGDEMVSLK and TGFBR1/TGFBR2, with PLDDT values exceeding 85, indicating a reliable interaction. To further investigate the specific mechanism of interaction between the core peptide YYTSASGDEMVSLK and TGFBR1/TGFBR2, PCR and WB confirmed that YYTSASGDEMVSLK upregulated the gene and protein expression of TGFBR1 and TGFBR2, increased SMAD2/3 expression and phosphorylation, and enhanced SMAD4 protein levels. Interestingly, YYTSASGDEMVSLK significantly increased TIMP1 levels and reduced the MMP1/TIMP1 ratio, though its inhibitory effect on MMP1 was minimal. Notably, YYTSASGDEMVSLK exhibited effects comparable to CCA in upregulating TGFβ-SMAD pathway molecules, but showed a superior trend in promoting collagen synthesis and regulating aging factors, this discrepancy may be attributed to the timing of administration, potentially missing the optimal time point for pathway molecule upregulation. In subsequent experiments, YYTSASGDEMVSLK was labeled with FITC and FLAG, WB analysis revealed that, upon incubation with YYTSASGDEMVSLK-Flag in BJ cells, the molecular weights of TGFBR1 and TGFBR2 increased, co-incubation with TGFBR1/TGFBR2 and FLAG antibodies resulted in overlapping bands, indicating binding of the peptide to TGFBR1 and TGFBR2.Super-resolution microscopy images indicated that YYTSASGDEMVSLK colocalized with TGFBR1 and TGFBR2 on the cell surface, further confirming its binding capability. In conclusion, YYTSASGDEMVSLK binds to and activates TGFBR2, particularly TGFBR1, thereby regulating the TGFβ-SMAD pathway, promoting collagen synthesis, and alleviating photoaging. Recent studies on CCA-derived peptides have shown that Luo et al. used commercially available CCA collagen peptide mixtures (CCACPs) in a UVB and D-galactose-induced aging mouse model. Through behavioral analysis and evaluation of biochemical indicators across multiple organs, they demonstrated that CCACPs systemically reduced oxidative stress and suppressed the expression of aging-related genes P16, P19, and P21, thus providing a comprehensive pharmacological assessment of the anti-aging effects of mixed peptides at the organismal level [54]. Wu et al. isolated CCA-derived low-molecular-weight peptide mixtures (CCAO) using enzymatic hydrolysis and filtration. In an epidermal-dermal co-culture model, they found that CCAO more significantly enhanced skin barrier function and type I/III collagen expression, while inhibiting MMP1 expression compared to CCA, confirming its protective effect on skin cells [55]. However, the CCA peptides used in the above studies were mixed oligopeptides that lacked further characterization by mass spectrometry. Moreover, both studies employed relatively simple models and did not thoroughly elucidate the underlying mechanisms, remaining limited to preliminary validation at the phenotypic and molecular levels. Building upon in vitro and in vivo aging models that confirmed the anti-photoaging effects of CCA via the TGFβ-SMAD/MMPs signaling pathway, this study employed mass spectrometry to identify low-molecular-weight bioactive peptides in CCA. Using PPI network analysis, we screened for peptides that directly interact with the TGFβ-SMAD pathway. The selected core peptide was synthesized using SPPS at a purity exceeding 95%. Further validation in a UV-induced photoaged fibroblast model showed that the peptide YYTSASGDEMVSLK simultaneously binds to and upregulates the expression of TGFBR1 and TGFBR2, thereby activating the TGFβ-SMAD pathway and promoting collagen synthesis. Thus, this study provides a more systematic and mechanistic understanding of the role of CCA and its core peptide YYTSASGDEMVSLK in improving photoaged skin. These findings provide new insights into the material basis and potential mechanisms of CCA in photoaging treatment, providing a theoretical foundation for the development of small-molecule bioactive peptides in the field of UV-induced skin aging.

## Conclusion

In conclusion, CCA and its active components demonstrate significant photoprotective effects. This study, for the first time, identified active peptides of CCA using mass spectrometry, enriched interacting proteins through PPI analysis with mouse proteins, and predicted biological processes involving CCA. These peptides were found to be closely related to genetic stability and protein translation. Validation through photoaging animal and cell models revealed that CCA significantly improved the appearance and structure of photoaged skin, increased dermal Collagen I content, reduced oxidative stress and inflammation, and decreased the expression of aging markers. Investigating the regulatory mechanism of CCA on collagen synthesis and degradation in photoaged skin, we found that CCA may promote collagen synthesis by activating the TGFβ-SMAD pathway and inhibit excessive collagen degradation by modulating the MMPs/TIMPs balance, effectively alleviating photoaging. Furthermore, this study confirmed that the core peptide YYTSASGDEMVSLK of CCA binds to and activates TGFBR1 and TGFBR2, thereby regulating the TGFβ-SMAD pathway, promoting collagen synthesis, and improving photoaging. Nevertheless, this study has limitations, as the post-binding modifications and activation mechanisms of CCA peptides with their targets require further investigation. In summary, this study confirms the pharmacological effects and mechanisms of CCA in delaying UV-induced skin aging, expanding its potential for development as an anti-photoaging agent.

## Supplementary Information

Additional file 1.

Additional file 2.

Additional file 3.

Additional file 4.

Additional file 5.

## Data Availability

The data will be available on request.

## Abbreviations

CCA: Colla Corii Asini
UV: Ultraviolet
ROS: Reactive oxygen species
ECM: Extracellular matrix
ESI: Electrospray ionisation
CUR: Curtain gas
CID: Collision-induced dissociation
FDR: False-discovery rate
PPI: Protein-Protein Interaction
MCODE: Molecular Complex Detection
SHG: Second-Harmonic Generation
TPEF: Two-photon excited fluorescence
HE: hematoxylin and eosin
IHC: Immunohistochemistry
WB: Western Blotting
SPPS: Solid-phase peptide synthesis
HPLC: High-performance liquid chromatography
TFA: Trifluoroacetic acid
IF: Immunofluorescence
SEM: Standard error of the mean
GO: Gene Ontology
KEGG: Kyoto Encyclopedia of Genes and Genomes
SN: Supernatant
CCACPs: CCA collagen peptide mixtures
