# Supplementary material for: Colla Corii Asini regulate collagen regeneration in UV exposure-induced skin photoaging in mice
Source: Chin Med. 2025 Sep 22;20:146. doi: 10.1186/s13020-025-01175-1 (PMC12452026; doi:10.1186/s13020-025-01175-1)
Supplement: Supplementary file 1 — Additional file 1. [file 13020_2025_1175_MOESM1_ESM.docx]

| **Gene** | **Primer sequences** |
| --- | --- |
| Il1a (Mouse) | FORWARD:GATTCTGAAGAAGAGAGACGGCTGAG  REVERSE:TCTGGTAGGTGTAAGGTGCTGATC |
| Il1b (Mouse) | FORWARD:GGACAGGATATGGAGCAACAAGTGG  REVERSE:TCATCTTTCAACACGCAGGACAGG |
| Il10 (Mouse) | FORWARD:GGACCAGCTGGACAACATAC  REVERSE:AGAGAAGCATGGCCCAGAAA |
| Tnf (Mouse) | FORWARD:GCTCCAGGCGGTGCTTGTTC  REVERSE:CCAGAGGGCTGATTAGAGAGAGAGGTC |
| Cdkn2a (Mouse) | FORWARD:TTCAGGTGATGATGATGGGCAACG  REVERSE:CGGGCGGGAGAAGGTAGTGG |
| Cdkn1a (Mouse) | FORWARD:CCTTGTGCCTGTCTTGCACTCTG  REVERSE:GCTGGTCTGCCTCCGTTTTCG |
| Trp53 (Mouse) | FORWARD:ACCGCCGACCTATCCTTACCATC  REVERSE:GGCACAAACACGAACCTCAAAGC |
| Col1a1 (Mouse) | FORWARD:ACAGGCGAACAAGGTGACAGAG  REVERSE:AGGAGAACCAGGAGAACCAGGAG |
| Tgfbr1 (Mouse) | FORWARD:CGTCGTCCGCAGCTCCTCTC  REVERSE:TGACTGAGACAAAGCAAAGACCATC |
| Tgfbr2 (Mouse) | FORWARD:GGAAGAATACACCACCAGCAGTC  REVERSE:GACACGGTAGCAGTAGAAGATGATG |
| Smad2 (Mouse) | FORWARD:GTCGTCCATCTTGCCATTCACTC  REVERSE:TCTTCCGTGTCCATTCTGCTCTCC |
| Smad3 (Mouse) | FORWARD:AGGGCTTTGAGGCTGTGTCTACC  REVERSE:TGCTGGTCAGTGTCTGTCTCC |
| Smad4 (Mouse) | FORWARD:TCCAGCCTTCCCATTTCCAATCATC  REVERSE:ATCCACATAGCCATCCACAGTCAC |
| Mmp1 (Mouse) | FORWARD:CAGTTGACAGGCTCCGAGAAATG  REVERSE:CACATCAGGGCACTCCACACTCTTG |
| Timp1 (Mouse) | FORWARD:GGATTCAAGGCTGTGGGGAATGC  REVERSE:ACTGCGGTTCTGGGACTTGTG |
| Gapdh (Mouse) | FORWARD:GCAAATTCAACGGGCACAGTCAAG  REVERSE:TCGCTCCTGGAAGATGGTGATG |
| IL1B (Human) | FORWARD:ATGGCTTATTACAGTGGCAATGAGG  REVERSE:AGTGGTTGTCGGAGATTCGTAG |
| TNF (Human) | FORWARD:CTCATCTACTCCCAGGTCCTCTTC  REVERSE:CGATGCGGGCTGATGGTGTG |
| CXCL8 (Human) | FORWARD:TTTTGCCAAGGAGTGCTAAA  REVERSE:CTGGGGTGGAACTTCTTAGA |
| TP53 (Human) | FORWARD:GCCCATCCTCACCATCATCACAC  REVERSE:GCACAAAACACGGCACCTCAAAGC |
| COL1A1 (Human) | FORWARD:AAAGATGGACTCAACGGTCTC  REVERSE:CATCGTGAGCCTTCTTCTTGAG |
| COL3A1 (Human) | FORWARD:CTCAGGGTGTCAAGGGTGAAAGTG  REVERSE:GTACCAGCCAGACCAGGAAGAC |
| TGFBR1 (Human) | FORWARD:CTCTTTCAAAAACTGGGTCTGTG  REVERSE:CATCAACATGAGTGAGATGCAG |
| TGFBR2 (Human) | FORWARD:GCCAACAACATCAACCCACAACAC  REVERSE:GCCACTGTCTCAAACTGCTCTG |
| CDKN2A (Human) | FORWARD:GGCCCGATCCAGGTCATGATG  REVERSE:ACCACCAGCGTGTCCAGGAAG |
| CDKN1A (Human) | FORWARD:GCCCCGTGAGCGATGGAACCTTC  REVERSE:CCTGCCTCCTTCCCAACTCATCC |
| HES1 (Human) | FORWARD:CGGCTAAGGTGTTTTGGAGGCTTC  REVERSE:CCGCTGTTGCTGGTGTAGACG |
| ID3 (Human) | FORWARD:GGACGACATGAACCCACTGCTACTC  REVERSE:GCTGTAGGATTTCCACCTGGCTAAG |
| IER2 (Human) | FORWARD:GAGCCAATGGACACGCAGGAG  REVERSE:GCTTTCTTGTCTCGGGACCAGTC |
| SMAD2 (Human) | FORWARD:CTCTTCTGGCTCAGTCTGTTAA  REVERSE:AAGGAGTACTTGTTACCGTCTG |
| SMAD3 (Human) | FORWARD:AGAGAGTAGAGACACCAGTTCT  REVERSE:GAAGTTAGTGTTTTTCGGGGATG |
| MMP1 (Human) | FORWARD:CACGCCAGATTTGCCAAGAGC  REVERSE:GCTTGACCCTCAGAGACCTTGG |
| MMP3 (Human) | FORWARD:GATTGGAGGTGACGGGGAAGC  REVERSE:TTCGGGATGCCAGGAAAGGTTC |
| MMP10 (Human) | FORWARD:CAGTTCCGCCTTTTCGCAAGATG  REVERSE:AGGATCACACTTGGCTGGCATC |
| MMP11 (Human) | FORWARD:CTGAGATCGACGCTGCCTTCC  REVERSE:GCCTTCCAGAGCCTTCACCTTC |
| MMP13 (Human) | FORWARD:CCCTTCCCAGTGGTGGGTGATG  REVERSE:TCTCGGAGCCTCTCAGTCATGG |
| MMP19 (Human) | FORWARD:GCTGACATCCGCCTCTCCTTC  REVERSE:CTGGGTTCTGTGGGGCACTGG |
| TIMP1 (Human) | FORWARD:CATCACTACCTGCAGTTTTGTG  REVERSE:TGGATAAACAGGGAAACACTGT |
| TIMP3 (Human) | FORWARD:GGCTGTGCAACTTCGTGGAGAG  REVERSE:AGCAAGGCAGGTAGTAGCAGGAC |
| MAPK14 (Human) | FORWARD:TCGCCGAAGGCACCATTCAAGTTC  REVERSE:TCCTGGCTGGAATCTAGCAGGTCTC |
| JUN (Human) | FORWARD:AAGAACTCGGACCTCCTCCACCTC  REVERSE:GCCCGTTGCTGGACTGGATTATC |
| FOS (Human) | FORWARD:TTCAGAGTTGACCAGTGTGGC  REVERSE:TGCTGCTGCTGCCCTTGCGG |
| GAPDH (Human) | FORWARD:ACACCCACTCCTCCACCTTTG  REVERSE:TCCACCACCCTGTTGCTGTAG |
| β-ACTIN (Human) | FORWARD:CTACCTCATGAAGATCCTGACC  REVERSE:CACAGCTTCTCCTTTGATGTCAC |
