# Supplementary material for: Colla Corii Asini regulate collagen regeneration in UV exposure-induced skin photoaging in mice
Source: Chin Med. 2025 Sep 22;20:146. doi: 10.1186/s13020-025-01175-1 (PMC12452026; doi:10.1186/s13020-025-01175-1)
Supplement: Supplementary file 3 — Additional file 3. [file 13020_2025_1175_MOESM3_ESM.docx]

HUMAN Sequence

Protein:Heat shock protein HSP 90-alpha

Gene name:HSP90AA1

MPEETQTQDQPMEEEEVETFAFQAEIAQLMSLIINTFYSNKEIFLRELISNSSDALDKIRYESLTDPSKLDSGKELHINLIPNKQDRTLTIVDTGIGMTKADLINNLGTIAKSGTKAFMEALQAGADISMIGQFGVGFYSAYLVAEKVTVITKHNDDEQYAWESSAGGSFTVRTDTGEPMGRGTKVILHLKEDQTEYLEERRIKEIVKKHSQFIGYPITLFVEKERDKEVSDDEAEEKEDKEEEKEKEEKESEDKPEIEDVGSDEEEEKKDGDKKKKKKIKEKYIDQEELNKTKPIWTRNPDDITNEEYGEFYKSLTNDWEDHLAVKHFSVEGQLEFRALLFVPRRAPFDLFENRKKKNNIKLYVRRVFIMDNCEELIPEYLNFIRGVVDSEDLPLNISREMLQQSKILKVIRKNLVKKCLELFTELAEDKENYKKFYEQFSKNIKLGIHEDSQNRKKLSELLRYYTSASGDEMVSLKDYCTRMKENQKHIYYITGETKDQVANSAFVERLRKHGLEVIYMIEPIDEYCVQQLKEFEGKTLVSVTKEGLELPEDEEEKKKQEEKKTKFENLCKIMKDILEKKVEKVVVSNRLVTSPCCIVTSTYGWTANMERIMKAQALRDNSTMGYMAAKKHLEINPDHSIIETLRQKAEADKNDKSVKDLVILLYETALLSSGFSLEDPQTHANRIYRMIKLGLGIDEDDPTADDTSAAVTEEMPPLEGDDDTSRMEEVD

Mouse Sequence

Protein:Heat shock protein HSP 90-alpha

Gene name:Hsp90aa1

MPEETQTQDQPMEEEEVETFAFQAEIAQLMSLIINTFYSNKEIFLRELISNSSDALDKIRYESLTDPSKLDSGKELHINLIPSKQDRTLTIVDTGIGMTKADLINNLGTIAKSGTKAFMEALQAGADISMIGQFGVGFYSAYLVAEKVTVITKHNDDEQYAWESSAGGSFTVRTDTGEPMGRGTKVILHLKEDQTEYLEERRIKEIVKKHSQFIGYPITLFVEKERDKEVSDDEAEEKEEKEEEKEKEEKESDDKPEIEDVGSDEEEEEKKDGDKKKKKKIKEKYIDQEELNKTKPIWTRNPDDITNEEYGEFYKSLTNDWEEHLAVKHFSVEGQLEFRALLFVPRRAPFDLFENRKKKNNIKLYVRRVFIMDNCEELIPEYLNFIRGVVDSEDLPLNISREMLQQSKILKVIRKNLVKKCLELFTELAEDKENYKKFYEQFSKNIKLGIHEDSQNRKKLSELLRYYTSASGDEMVSLKDYCTRMKENQKHIYFITGETKDQVANSAFVERLRKHGLEVIYMIEPIDEYCVQQLKEFEGKTLVSVTKEGLELPEDEEEKKKQEEKKTKFENLCKIMKDILEKKVEKVVVSNRLVTSPCCIVTSTYGWTANMERIMKAQALRDNSTMGYMAAKKHLEINPDHSIIETLRQKAEADKNDKSVKDLVILLYETALLSSGFSLEDPQTHANRIYRMIKLGLGIDEDDPTVDDTSAAVTEEMPPLEGDDDTSRMEEVD
