# Supplementary figures and images for: Colla Corii Asini regulate collagen regeneration in UV exposure-induced skin photoaging in mice
Source: Chin Med. 2025 Sep 22;20:146. doi: 10.1186/s13020-025-01175-1 (PMC12452026; doi:10.1186/s13020-025-01175-1)

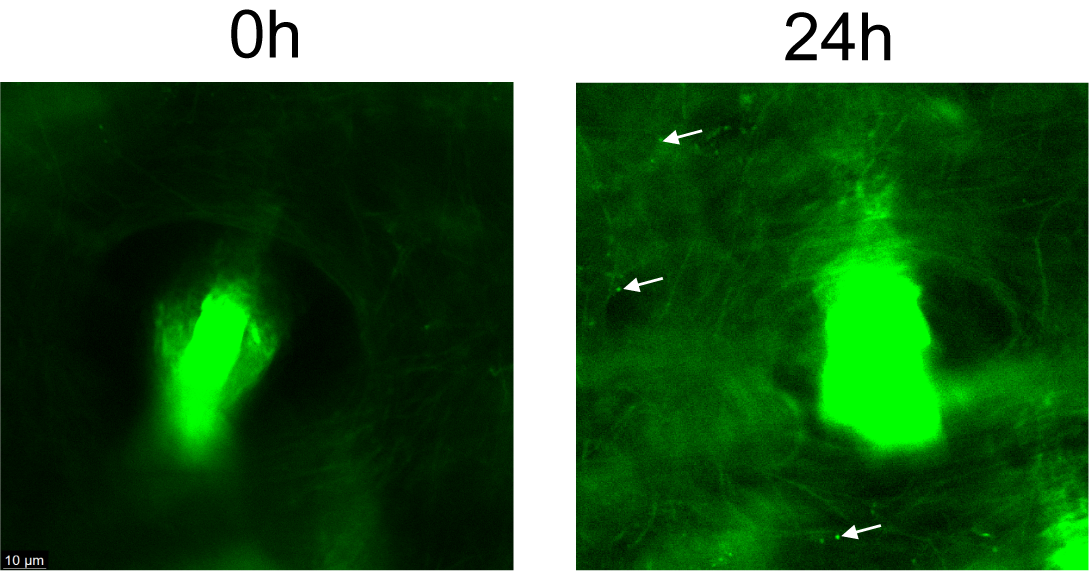

Supplement: Supplementary file 4 — Additional file 4. [file 13020_2025_1175_MOESM4_ESM.tif]

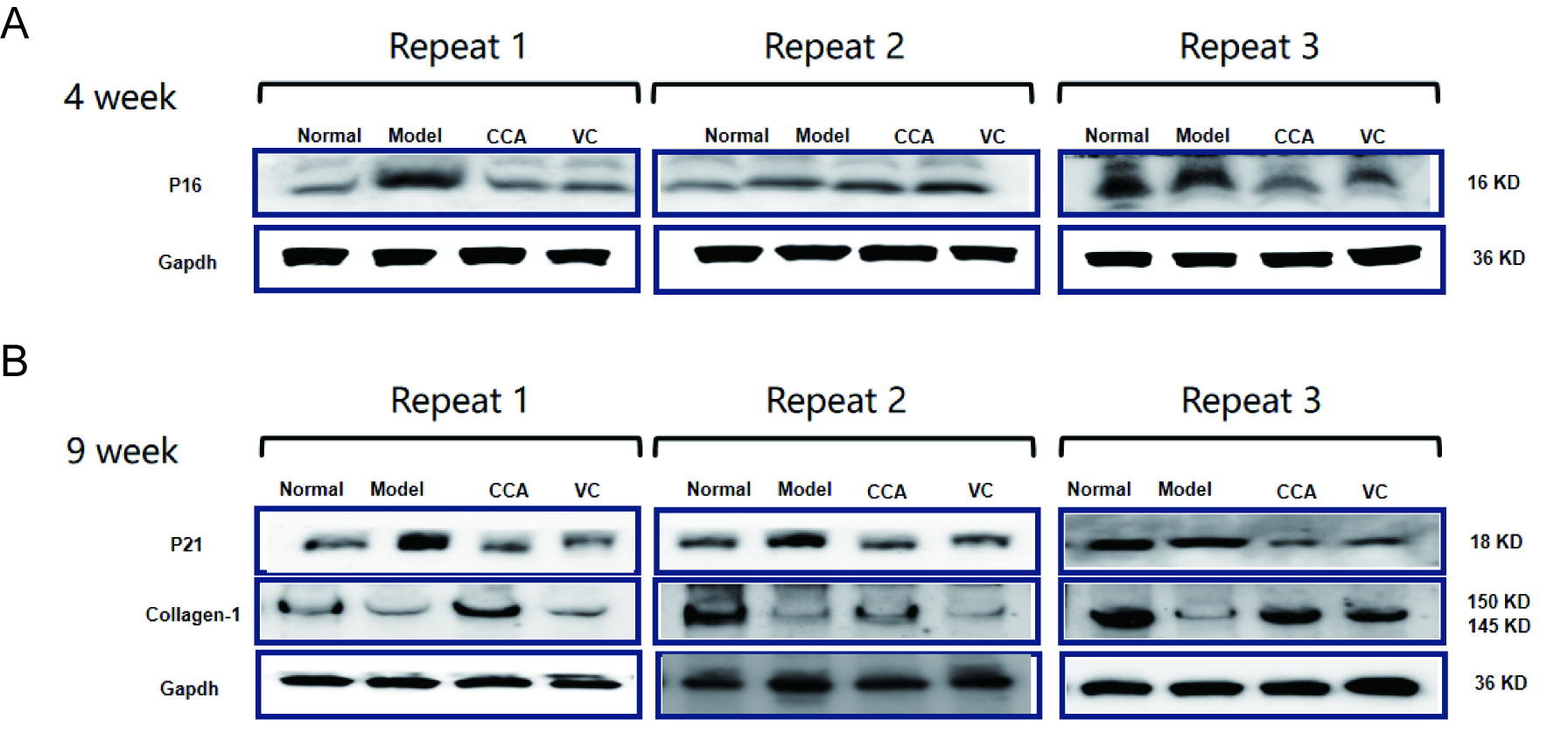

Supplement: Supplementary file 5 — Additional file 5. [file 13020_2025_1175_MOESM5_ESM.tif]
